# MXene‐Supported Single‐Atom Electrocatalysts

**DOI:** 10.1002/advs.202414674

**Published:** 2025-03-27

**Authors:** Jianan He, Joshua D. Butson, Ruijia Gu, Adrian Chun Minh Loy, Qining Fan, Longbing Qu, Gang Kevin Li, Qinfen Gu

**Affiliations:** ^1^ Department of Chemical Engineering The University of Melbourne Parkville VIC 3010 Australia; ^2^ Australian Synchrotron ANSTO 800 Blackburn Rd Clayton VIC 3168 Australia

**Keywords:** catalytic applications, MXenes, single‐atom catalysts, support, synthesis and characterization

## Abstract

MXenes, a novel member of the 2D material family, shows promising potential in stabilizing isolated atoms and maximizing the atom utilization efficiency for catalytic applications. This review focuses on the role of MXenes as support for single‐atom catalysts (SACs) for various electrochemical reactions, namely the hydrogen evolution reaction (HER), oxygen evolution reaction (OER), oxygen reduction reaction (ORR), carbon dioxide reduction reaction (CO_2_RR), and nitrogen reduction reaction (NRR). First, state‐of‐the‐art characterization and synthesis methods of MXenes and MXene‐supported SACs are discussed, highlighting how the unique structure and tunable functional groups enhance the catalytic performance of pristine MXenes and contribute to stabilizing SAs. Then, recent studies of MXene‐supported SACs in different electrocatalytic areas are examined, including experimental and theoretical studies. Finally, this review discusses the challenges and outlook of the utilization of MXene‐supported SACs in the field of electrocatalysis.

## Graphical abstract

This review discusses the characteristics and synthesis of MXenes to elucidate their benefits as support materials. Next, methods and characterization for anchoring SAs onto MXene are discussed. Subsequently, a comprehensive discussion is provided on the recent applications of MXene‐supported SACs in electrocatalysis. Finally, the future challenges and prospects of MXene‐supported SACs for electrocatalysis are outlined.

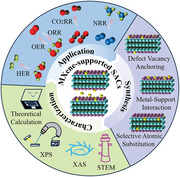

## 1 Introduction

Fossil fuels are primary energy sources widely used in many aspects of modern life, which, however, results in significant emissions of greenhouse gases.^[^ ^1^ ^]^ The development of renewable energy and CO_2_ transformation technologies plays a vital role in realizing a carbon‐neutral landscape.^[^ ^2^ ^]^ Among current methods, electrocatalysis offers a sustainable and efficient approach to producing green fuels and reducing CO_2_ into valuable products.^[^ ^3^ ^]^ For instance, water electrolysis, involving splitting water into hydrogen and oxygen, is crucial for green hydrogen production.^[^ ^4^ ^]^ With promising and tunable catalysts, the electrolysis process can be achieved at low overpotential, realizing high energy efficiency. Additionally, a suitable catalyst can help to improve the intrinsic activity and Faradaic efficiency toward producing NH_3_.^[^ ^5^ ^]^ The electrocatalytic CO_2_ reduction process, transforming CO_2_ into gas or liquid value‐added carbon‐based products, promotes the recycling of CO_2_ as a carbon‐neutral feedstock.^[^ ^6^ ^]^ The electrochemical activity and selectivity of CO_2_ conversion to specific products are normally realized by engineering the electrocatalysts and the microenvironment between catalysts and electrolytes.^[^ ^7^ ^]^ During these electrocatalysis processes, including the hydrogen evolution reaction (HER), oxygen evolution reaction (OER), oxygen reduction reaction (ORR), and carbon dioxide reduction reaction (CO_2_RR), catalysts play important roles in reaction performance and product rates. It is highly desirable to pursue endeavors in catalyst design and investigate novel materials and structures to alleviate the kinetic limitations observed in electrocatalytic processes.

The key goals in this field are to enhance the efficiency and performance of catalysts for these reactions across a range of applications. Accordingly, significant efforts have been devoted to developing novel catalysts, such as Pt group metals,^[^ ^8^ ^]^ metal carbides,^[^ ^9^ ^]^ metal nitrides,^[^ ^10^ ^]^ metal oxides,^[^ ^11^ ^]^ metal sulfides^[^ ^12^ ^]^ and metal phosphides.^[^ ^13^ ^]^ Among them, platinum (Pt) is well known as the most effective catalyst for the HER and ORR,^[^ ^8a,b^ ^]^ while ruthenium (Ru) possesses outstanding activity as an OER catalyst.^[^ ^8c–e^ ^]^ They also show outstanding stability under strong acidic conditions. However, the application of precious metal catalysts is hindered by limited abundance and high costs, hence the atom efficiency needs to be improved. Alternatively, efficient non‐noble metal catalysts are being increasingly employed to provide cost‐competitive performance due to the low cost and rapid transfer of electrons.^[^ ^14^ ^]^ There are still some challenges in the development of non‐precious catalysts. One is lower activity than noble metals, leading to limited efficiency and increased energy consumption.^[^ ^15^ ^]^ Another is the insufficient stability of these catalysts, and elements might leach and aggregate during the reaction.^[^ ^15^, ^16^ ^]^ To address these issues, size reduction of active catalysts is proven to be an effective strategy to maximize atom efficiency and maintain good catalytic performance.

Single‐atom catalysts (SACs), a class of material with mononuclear metal complexes anchored on supports, have provided novel insights into creating economical and high‐performance catalysts. Downsizing nanoparticles (above 1 nm)^[^ ^17^ ^]^ into nanoclusters (several to hundreds of atoms),^[^ ^18^ ^]^ and even single atoms (SAs) can improve the atomic dispersion and maximize atomic efficiency for both noble and non‐noble metal catalysts.^[^ ^19^ ^]^ Zhang et al. studied the impact of size on Nb‐in‐C through theoretical and experimental approaches. By density functional theory (DFT) calculation, they showed that the electrons on the niobium SAs incorporated into the graphitic layers, leading to increased density of states (DOS) near the Fermi surface. According to further analysis of DOS, it is demonstrated that niobium SAs had much higher values than larger niobium particles (a niobium‐terminated {111} surface and bulk niobium). This means the niobium SAs has superior catalytic potential than niobium nanoparticles. Experimentally, the ORR property of niobium SAs‐in‐C and the niobium nanoparticle‐in‐C were tested in an O_2_‐saturated 0.1 M KOH solution. The catalyst with niobium SAs shows a higher kinetic‐limiting current density (12.3 mA cm^−2^ at −0.5 V) and quicker reaction pathway (4e^−^) than the catalyst with niobium particles (0.912 mA cm^−2^ at −0.5 V, 2e^−^), which means a better catalytic activity.^[^ ^20^ ^]^ Additionally, in comparison to nanoclusters and nanoparticles, SAs exhibit unique active sites, consistent activity at each catalytic center, tunable morphology, and flexible electronic structures, which leads to a lower energy barrier of process and adjustable selectivity for various reactions.^[^ ^21^ ^]^ There are many approaches to designing and constructing SACs, including wet chemistry,^[^ ^22^ ^]^ atomic layer deposition,^[^ ^23^ ^]^ and electrochemical deposition.^[^ ^24^ ^]^ Different types of materials are employed as supports for anchoring SAs, such as carbon materials,^[^ ^25^ ^]^ metal oxides,^[^ ^26^ ^]^ metal‐organic frameworks,^[^ ^27^ ^]^ and zeolites.^[^ ^28^ ^]^ The catalytic activity, selectivity, and mass loading of SACs are strongly influenced by the interaction and coordination between metal atoms and neighboring non‐metallic atoms of the support.^[^ ^29^ ^]^ Additionally, due to the high surface energy of SAs, they are prone to agglomeration during catalytic processes.^[^ ^30^ ^]^ Therefore, developing SACs with outstanding catalytic stability is another crucial aspect of their industrial applications. So far, constructing strong metal‐support interactions (SMSIs) has been the primary strategy for synthesizing stable SACs. The strong surface interactions effectively help prevent aggregation and modify the electronic structure of electroactive sites to further enhance the catalytic stability of SAs through electronic metal‐support interactions. Overall, suitable support is critical for efficient and stable SACs.^[^ ^31^ ^]^

Since the discovery of graphene, 2D materials have attracted great attention as a powerful platform to support SAs, also including graphitic carbon nitride (g‐C_3_N_4_), metal carbides (e.g., Mo_2_C, WC, and MXenes), and transition metal dichalcogenides (TMDs).^[^ ^32^ ^]^ Compared with 3D catalysts, 2D materials provide several advantages due to their unique morphology, large specific surface area, numerous coordination‐unsaturated sites, abundant intrinsic defects, and tunable electronic structure.^[^ ^33^ ^]^ As a new type of 2D material, the 2D configuration of MXenes, comprising diverse forms of transition metal carbides, nitrides, and carbonitrides, demonstrates the potential to serve as a supporting platform for SAs. Besides the general advantages of 2D materials, MXenes possess many unique properties including tunable surface chemistry, excellent electronic conductivity, high hydrophilicity, and good mechanical properties. Unlike 2D metal oxides and g‐C_3_N_4_, graphene and MXenes can be highly conductive.^[^ ^34^ ^]^ In particular, MXene‐supported catalysts show superior charge transfer kinetics compared to those supported by reduced graphene oxide (rGO). This is due to the fact that the properties of MXenes can be more easily regulated through functionalization, alloying, and adjusting the chemical composition, resulting in enhanced interfacial and electronic interactions between SAs and MXenes.^[^ ^35^ ^]^ Specifically, for anchoring SAs, MXenes have intrinsically electronegative surface functional groups that can adsorb cations and provide active sites. The inherent defects created during the etching process can also be used as sites to introduce heteroatoms.^[^ ^36^ ^]^

This review begins by examining the characteristics and synthesis techniques of MXenes to elucidate the benefits of MXene support materials. Next, methods for anchoring SAs onto MXene surfaces are explored and the sophisticated characterization techniques used to study isolated atoms on MXenes are discussed. Subsequently, a comprehensive discussion is provided on the recent applications of MXene‐supported SACs in electrocatalysis. Finally, the future challenges and prospects of MXene‐supported SACs for electrocatalysis are outlined.

## 2 MXene‐Supported Single‐Atom Materials

### 2.1 Characteristics of MXenes as Supporting Substrates

**Figure 1:**
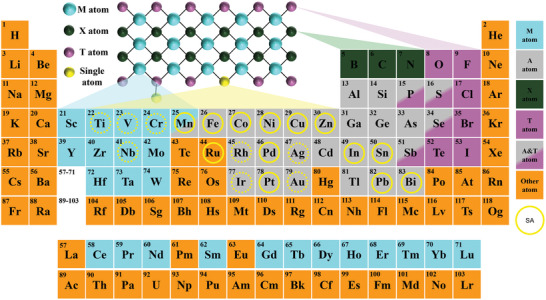
The periodic table shows elements involved in the formation of MAX phases and MXenes in previous publications. Light blue: M atoms; gray: A atoms; dark green: X atoms; purple: (T) elements; solid circles: SAs discussed in the present work with experiments; dotted circles: SAs discussed in the present work with theoretical calculations.

Throughout the synthesis of MXene‐supported SACs, support materials play a vital role in offering mechanical stability, enhancing atomic‐active site efficiency, and accelerating charge transfer kinetics.^[^ ^37^ ^]^ Therefore, an ideal support material must exhibit several key properties, including the ability to prevent atom aggregation, high conductivity to facilitate electron transfer between the doped atoms and the support, strong mechanical and electrochemical stability during oxidation‐reduction reactions, and good hydrophilicity.^[^ ^38^ ^]^ MXenes, 2D transition metal carbides and nitrides, are new additions to the family of 2D materials. Generally, MXenes can be synthesized by selectively etching a precursor of nanolaminate materials (MAX phases).^[^ ^39^ ^]^ The general formula of MAX phases can be written as M*_n_*_+1_AX*_n_* (*n* = 1–4), where M represents a left‐side transition metal (TM), A represents a right‐side TM or p‐block element, and X denotes carbon and/or nitrogen. The A atoms are etched to produce a MXene (M*_n_*_+1_X*_n_* T_x_) with T surface functional groups, where T depends on the etching method. **Figure** **1** illustrates the compositions of MAX, MXenes, and MXene‐supported SAs. MXenes have drawn considerable attention as a promising support material with the unique properties of high conductivity, abundant vacancies for dopant atoms, and high redox activity.^[^ ^40^ ^]^

First, the electronic structure, surface functional groups, and other properties can be manipulated through various synthetic conditions. Compared to other support materials such as graphene, MXenes possess easily adjustable electronic energy levels, charge mobility, and conductivity with a unique sandwich structure (X as the central layer and M as the outer layers, surrounded by terminal groups).^[^ ^41^ ^]^ The electronic properties of MXenes mainly depend on the outer metal layer and surface functionalized substituents. MXenes can be metalloids, semiconductors, topological insulators, or high insulators depending on the M metal and terminal groups.^[^ ^42^ ^]^ For instance, the SMSI can be tunable. When the A atoms are substituted with surface groups such as ‐F, ‐Cl, ‐O, or ‐OH, they receive one or two electrons from each M metal atom. This results in the formation of a new bonding orbital by hybridizing the p and d orbitals of the M atoms, which ultimately leads to a reduction in the Fermi level. This is more difficult to achieve for other 2D materials.^[^ ^43^ ^]^ Typically, MXenes have higher electrical conductivity compared to other 2D materials (10–100 S cm^−1^),^[^ ^44^ ^]^ where Ti‐based MXenes have demonstrated conductivities of up to 24 000 S cm^−1^.^[^ ^45^ ^]^ Due to the direction‐dependent nature of electrons and holes, the charge mobility of MXenes is anisotropic, with greater conductivity within the plane than perpendicular to it.^[^ ^46^ ^]^

Second, the surface of MXene layers is usually chemically stable, although carbide MXenes are generally more stable than nitride MXenes.^[^ ^47^ ^]^ Also, the stability of MXenes is typically determined by the terminal groups. Generally, the ‐F group with weak chemical bonding is prone to substitution by other groups; the ‐OH group easily decomposes. MXenes with the ‐O groups are the most stable, which benefits catalyst application in water electrolysis.^[^ ^48^ ^]^ The remarkable stability of MXenes helps them to withstand harsh environments during the synthesis of MXene‐supported SACs and the challenging electrochemical reaction conditions.

Third, MXenes exhibit excellent hydrophilicity because of the abundant surface groups such as ‐F, ‐Cl, ‐O, and ‐OH.^[^ ^49^ ^]^ Gas products generated on the surface of electrocatalysts can obstruct further reactions and reduce the real active sites.^[^ ^50^ ^]^ It is important to accelerate bubble separation from the electrode surface to boost catalytic activity. Water molecules are attracted to hydrophilic surfaces, which help to release gas bubbles and improve performance. Therefore, hydrophilicity is considered a key factor in optimizing catalysts.^[^ ^51^ ^]^

The distinctive characteristics of MXenes render them appropriate as support materials for SACs. With its 2D structure, high electrical conductivity, large surface area, tunable surface chemistry, and chemical resilience, MXenes are proven to be an adaptable material for such applications. Consequently, understanding the synthesis of MXenes contributes to the future development of MXene‐supported materials tailored for specific applications.

### 2.2 Synthesis Routes Toward MXenes

SAs are anchored on substrates by coordination bonds, so they can be designed with a specific coordination environment for the target process.^[^ ^52^ ^]^ MXenes can be synthesized by removing the A atoms in the MAX phase, which generates suspended bonds on the surface of M atoms. The surface terminal groups and morphology of MXenes are controlled by various synthesis methods, impacting the ability to immobilize SAs. Therefore, selecting an appropriate etching method is crucial. To further elucidate the advancement synthesis method, chronological progress over the last two decades is discussed as follows.

#### 2.2.1 Fluoride‐Based Etching Methods

**Figure 2:**
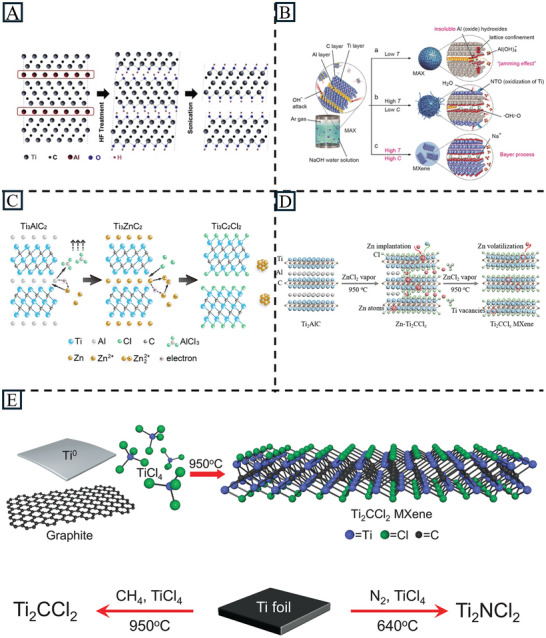
A) Schematic of the exfoliation process for Ti_3_AlC_2_ with HF. Reproduced with permission.^[^ ^53^ ^]^ Copyright 2011, Wiley‐VCH. B) The reaction between Ti_3_AlC_2_ and aqueous NaOH solution under different conditions. Reproduced with permission.^[^ ^60^ ^]^ Copyright 2018, Wiley‐VCH. C) Schematic of the etching process for Ti_3_AlC_2_ with molten salt. Reproduced with permission.^[^ ^61^ ^]^ Copyright 2019, American Chemical Society. D) Schematic illustration of the etching of Ti_2_AlC toward vacancy‐enriched Ti_2_CCl_x_ MXene. Reproduced with permission.^[^ ^62^ ^]^ Copyright 2024, Wiley‐VCH. E) Schematic diagram of the synthesis by bottom‐up method. Reproduced with permission.^[^ ^65^ ^]^ Copyright 2023, The American Association for the Advancement of Science.

In 2011, the first MXene, Ti_3_C_2_T_x_ (T = ‐OH, ‐F), was synthesized by Naguib et al. by removing Al from Ti_3_AlC_2_ in hydrofluoric acid (HF) at room temperature (**Figure** **2**A).^[^ ^53^ ^]^ MXenes etched with HF typically exhibit multi‐layer morphology, which can be then delaminated into single‐ or few‐layer flakes.^[^ ^54^ ^]^ In 2014, Ghidiu et al. proposed a safer, less toxic pathway to synthesize MXenes by generating HF in situ using fluoride salts (e.g., LiF) with HCl.^[^ ^55^ ^]^ This process allows the simultaneous intercalation of Li^+^ cations, increasing c‐lattice parameters and enabling exfoliation into few‐layered MXenes through sonication and centrifugation. Nonetheless, these approaches with HF directly or indirectly face challenges including health hazards from HF, limited applicability to Al‐containing MAX phases, and instability of ‐F for atom doping. Consequently, it is crucial to explore alternative etching methods that are F‐free.

#### 2.2.2 Fluoride‐Free Etching Methods

In addition to the environmental benefits and operational safety, fluoride‐free approaches aim to expand the types of MXenes,^[^ ^56^ ^]^ boost conductivity,^[^ ^57^ ^]^ enhance energy storage capacity,^[^ ^58^ ^]^ and optimize the electrochemical activity^[^ ^59^ ^]^ for various applications. Each method has advantages and disadvantages, and some typical fluoride‐free approaches are discussed in detail here.

Theoretically, alkalis are a good choice for etching Al‐containing MAX phases due to their strong reactivity with Al. Li et al. prepared high‐purity multilayer MXene via an alkali‐assisted hydrothermal method. Ti_3_AlC_2_ was soaked in 27.5 M NaOH at 270 °C to produce high purity (92%) multilayer Ti_3_C_2_T_x_ (T = O, OH) (Figure 2B).^[^ ^60^ ^]^ However, the dangers of using highly concentrated alkali at high temperatures hinder its application at large scale.

Li et al. first used molten salt ZnCl_2_ to synthesize Zn‐containing MAX phases and ‐Cl terminated MXenes (Figure 2C). This opened the door to explore hard‐to‐obtain MAX‐phases via common powder metallurgy and modify MXenes with ‐Cl surface groups.^[^ ^61^ ^]^ Lewis acid etching techniques were extended by another group to other MAX phases, expanding the range of precursors available (A = Al, Si, Ga) and enabling control of surface groups (‐Cl, ‐Br, ‐I, ‐S, ‐Se, and ‐NH) for tailored surface chemistry in specific applications.^[^ ^59^ ^]^ Recently, Guo et al. even synthesized the MXene Ti_2_CCl_x_ with metal chloride (ZnCl_2_) vapor within 5 min (Figure 2D).^[^ ^62^ ^]^ Overall, this approach expands the potential applications of molten salt etching techniques. Nevertheless, the resulting MXenes predominantly exhibit accordion‐like structures, requiring further investigation to better support doped atoms.

#### 2.2.3 Bottom‐Up Synthesis of MXenes

In addition to chemical etching of MAX or non‐MAX phases, some bottom‐up synthetic routes bypassing the MAX phase can also be employed to produce MXenes. The bottom‐up methods to synthesis large‐area MXenes assembles small molecules into ordered 2D layered structures via crystal growth.^[^ ^63^ ^]^ By building the material from atomic or molecular components, it allows for more precise control over the material properties. Recently, Wang et al. successfully grew MXene sheets oriented perpendicular to the substrate by direct chemical vapor deposition (CVD) synthesis. Metals (Ti or Zr foil) were exposed to metal halides (TiCl_4_, ZrCl_4_ or ZrBr_4_) and an “X” source (CH_4_ or N_2_) atmosphere to produce MXenes (Ti_2_CCl_2_, Ti_2_NCl_2_, Zr_2_CCl_2,_ and Zr_2_CBr_2_) at the temperature above 640 °C. This method not only offers the opportunity for new MXenes (Zr_2_CCl_2_ and Zr_2_CBr_2_), but also diversifies the morphology from carpet‐like to flower‐like structures. Special morphology exposes fresher surface and edge sites with high catalytic activities, improving accessibility for ion intercalation and chemical transformations which are good for future modifications and energy storage applications. The same group also demonstrated the direct synthesis of MXenes via sealing a mixture of Ti, carbon source (graphite), and TiCl_4_ in a quartz ampoule and heating it at high temperatures (950 °C) for 2 h. This is more efficient than traditional etching methods (24 h for in situ HF etching),^[^ ^64^ ^]^ while the prepared MXene showed a high degree of structural perfection with sufficient ‐Cl coverage of the surface (Figure 2E).^[^ ^65^ ^]^

Besides the typical methods mentioned above, several other techniques are also used for MXene synthesis, including etching with fluoride‐based salts,^[^ ^66^ ^]^ mechanical milling,^[^ ^67^ ^]^ and etching with halogens.^[^ ^68^ ^]^ Despite numerous methods for MXenes preparation, several MXene variants remain largely theoretical. Moreover, single‐layer production predominantly depends on the LiF + HCl or organic base intercalation (e.g., cetyl trimethyl ammonium bromide cation,^[^ ^69^ ^]^ tetramethylammonium hydroxide^[^ ^70^ ^]^). Hence, improving and innovating new synthesis techniques for MXenes holds significant promise and is crucial for applications as a substrate. **Table** **1** compares surface terminal groups and the structure of MXenes generated via various etching methods.

**Table 1:** MXenes generated via various synthesized methods.

Methods	MXene type	Functional group	Structure	Refs.
HF etching	Ti_3_C_2_T_x_ Zr_3_C_2_T_x_	‐O, ‐OH, ‐F	multi‐layer, single layer	[54, 71]
In situ HF etching	Ti_3_C_2_T_x_	‐O, ‐OH, ‐F	few‐layer, single layer	[55]
Fluoride‐based salts etching	Ti_3_C_2_T_x_	‐O, ‐F, ‐N	multi‐layer,	[66]
Alkali etching	Ti_3_C_2_T_x_	‐O, ‐OH	multi‐layer, single layer	[60]
Mechanical milling	Ti_3_C_2_T_x_	‐O, ‐OH	porous layer	[67]
Etching with halogens	Ti_3_C_2_T_x_	‐I, ‐Br	monolayer,	[68]
Molten salt etching	Ti_3_C_2_T_x_ Ti_2_CT_x_	‐O, ‐Cl, ‐S, ‐Se, and ‐NH	accordion‐like structures	[59, 61]
CVD	Ti_2_CCl_2_ Ti_2_NCl_2_ Zr_2_NCl_2_ Zr_2_CBr_2_	‐Cl, ‐Br	carpet‐like structures, flower‐like structures	[65]
Direct synthesis	Ti_2_CCl_2_ Ti_2_NCl_2_	‐Cl	stack‐like structures	[65]

### 2.3 Synthesis of MXene‐Supported SAs

**Figure 3:**
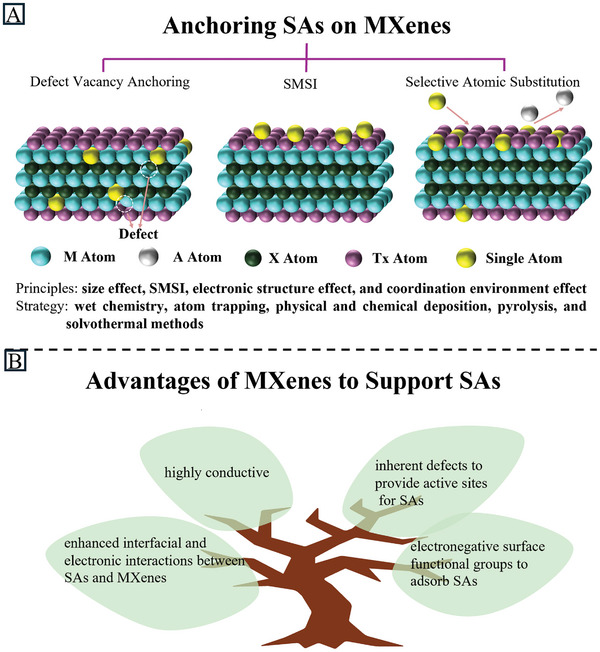
A) Three methods for anchoring single metal atoms on MXenes; B) The advantages of MXenes to support SAs.

Because of their high surface energy, SAs tend to aggregate during synthesis and electrochemical reactions.^[^ ^19a^ ^]^ Synthesis strategies based on principles including size effect, SMSI, electronic structure effect, and coordination environment effect, are vital in the development of SACs.^[^ ^72^ ^]^ There are several strategies to synthesize SACs, including wet chemistry,^[^ ^73^ ^]^ atom trapping methods,^[^ ^74^ ^]^ physical and chemical deposition,^[^ ^24^ ^]^ pyrolysis,^[^ ^75^ ^]^ and solvothermal methods.^[^ ^76^ ^]^ These common preparation methods are often combined with specific strategies to achieve atomic dispersion of metals with high loading, including defect engineering, ligand modification, and spatial confinement.^[^ ^77^ ^]^ Due to the advantages of MXenes as support materials, these techniques are employed to introduce precursors onto the MXene surface and transform them into SAs to synthesize MXene‐supported SACs (**Figure** **3**). The approach for incorporating SAs into MXenes significantly influences the performance of the catalysts for particular applications.

#### 2.3.1 Defect Vacancy Anchoring

Defect engineering is an effective method to enhance the interaction between SAs and the substrate, which benefits the synthesis of SACs with high metal content and good stability. It has been proven that individual atoms can be fixed and stabilized by modified support via pinning at electronic or structural defects associated with coordinatively unsaturated sites.^[^ ^78^ ^]^ Therefore, the concentration and stability of metal SAs intrinsically depend on the density and stability of defects. The removal of A atoms from MAX phases results in atomic vacancies such as metal vacancies and carbon vacancies in MXenes. With the introduction of surface functional groups, there may also be some vacancies like O‐vacancies due to non‐uniform distribution. These intrinsic defects have high reducing activity and provide promising anchoring sites for SAs.

Zhang et al. synthesized Mo_2_TiC_2_T_x_‐Pt_SA_ with Pt SAs immobilized by Pt‐C bonds on Mo vacancies, which were introduced by electrochemical exfoliation. DFT calculations show that Pt SAs with positive charge comprise the adsorption position for H^+^ to benefit the HER.^[^ ^79^ ^]^ In addition to metal atom vacancies such as *V*_Mo_ and *V*_Ti_, oxygen vacancy (*V*_O_) is also generated from the transformation of surface groups. Zhang et al. designed Pt SACs on monolayer Ti_3_C_2_T_x_ by rapid thermal shock under an H_2_ atmosphere. The Pt_SA_ anchoring on the *V*_O_ of Ti_3_C_2_T_x_ achieved a Gibbs free energy of 0.02 eV and showed outstanding catalytic activity.^[^ ^80^ ^]^ With the defect vacancy strategy, Ni,^[^ ^81^ ^]^ Fe,^[^ ^82^ ^]^ Cu, Co, Mn, Zn, In, Sn, Pb, and Bi SAs^[^ ^83^ ^]^ were also successfully anchored on MXenes.

#### 2.3.2 Strong Metal‐Support Interaction

Besides anchoring on vacant sites, SAs can also be immobilized on supports with specific sites by strong metal‐support interaction. Ramalingam et al. fixed Ru SAs on Ti_3_C_2_T_x_ through nitrogen and sulfur heteroatom dopants.^[^ ^84^ ^]^ A mixture of Ti_3_C_2_T_x_, RuCl_3_·xH_2_O, and thiourea was freeze‐dried and annealed at high temperatures. Ru SAs first interact with or are adsorbed on Ti_3_C_2_T_x_ by ‐O and ‐OH groups and are then stabilized with N and S dopants. Strong Ru‐N (O) and Ru‐S interactions were observed with XAFS, demonstrating electronic coupling between Ru_SA_ and the MXene via N and S atoms. This strategy has been effectively applied to Pd SAs as well, which were fixed on Ti_3_C_2_T_x_ via ‐O groups.^[^ ^85^ ^]^

#### 2.3.3 Selective Atomic Substitution

Due to the different redox activity of different transition metals (TMs), SAs can be introduced onto MXenes by selectively replacing surface atoms. Kuznetsov et al. developed a synthetic method to introduce Co SAs into Mo_2_CT_x_ by Co‐substitution. Initially, Co was intercalated into Mo_2_Ga_2_C, followed by the elimination of Ga through an HF etching procedure. The introduction of the Co dopant onto the MXene not only generated an additional reaction site but also modified the surface adsorption energetics, thereby impacting catalytic reaction kinetics.^[^ ^86^ ^]^

## 3 Characterization of MXene‐Supported SAs

A critical challenge concerning MXene‐supported SACs involves characterizing the presence, distribution, and local geometric and electronic structure of SAs, as well as changes to SAs during reactions. Currently, numerous advanced techniques have emerged to study SAs at the atomic level, including high‐angle annular dark‐field scanning transmission electron microscopy (HAADF‐STEM), synchrotron X‐ray absorption spectroscopy (XAS), X‐ray photoelectron spectroscopy (XPS), Raman spectroscopy, theoretical calculations, and many others. Furthermore, these techniques are extended to in situ characterization for studying SACs during reactions and providing detailed insights into reaction kinetics, reaction mechanisms, and active sites.

### 3.1 High‐Resolution Electron Microscopy

**Figure 4:**
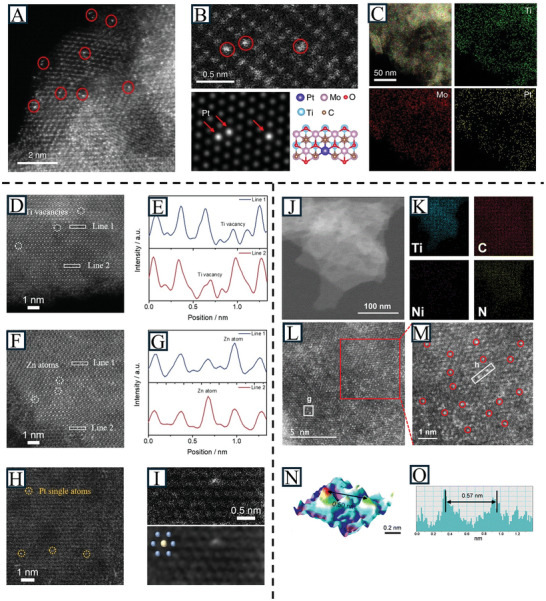
A) HAADF–STEM image of Mo_2_TiC_2_T_x_–Pt_SA_. B) Magnified HAADF–STEM image of Mo_2_TiC_2_T_x_–Pt_SA_ and its corresponding simulated image, and illustration of the structure of Mo_2_TiC_2_T_x_–Pt_SA_, showing the isolated Pt atoms (circled). C) STEM–EDS elemental mapping of Mo_2_TiC_2_T_x_–Pt_SA_ nanosheets. Reproduced with permission.^[^ ^79^ ^]^ Copyright 2018, Springer Nature. D) Atomic‐resolution HAADF‐STEM image of Ti_2_CCl_x_ MXene. E) Line intensity profiles obtained from the rectangular regions in (D), showed the existence of Ti vacancies. F) Atomic‐resolution HAADF‐STEM image of Zn–Ti_2_CCl_x_. G) Line intensity profiles obtained from the rectangular regions in (F), showed the existence of single‐atom Zn. H) Atomic‐resolution HAADF‐STEM image, and I) the experimental and simulated HAADF‐STEM images of Ti_2_CClx–Pt_SA_. Reproduced with permission.^[^ ^62^ ^]^ Copyright 2024, Wiley‐VCH. J) HAADF‐STEM image of Ni_SA_@N‐Ti_3_C_2_T_x_. K) Corresponding EDS analysis. L) and M) Atomically resolved HAADF‐STEM images. N) 3D atom‐overlapping Gaussian‐function fitting mapping for the g area in (L) illustrating the atomic structure and arrangement of Ni_SA_@N‐Ti_3_C_2_T_x_. O) Corresponding profiles of the h area in (M). Reproduced with permission.^[^ ^87^ ^]^ Copyright 2024, The Royal Society of Chemistry.

The development of advanced electron microscopes provides direct evidence of nanoparticles or even SAs. By employing various electron microscopy techniques, such as aberration‐corrected high‐angle annular dark‐field scanning transmission electron microscopy (AC‐HAADF‐STEM), the location of SAs can be observed through bright and dark images. It also provides information about the coordination states of SAs, serving as a starting model for theoretical calculations. For instance, Zhang et al. demonstrated the successful formation of Pt_SA_ on Mo_2_TiC_2_T_x_ with STEM. The bright spots observed in the HAADF‐STEM represent heavy atoms (Pt) (**Figure** **4**A–C).^[^ ^79^ ^]^ Guo et al. investigated the sites of SAs (Ti vacancies) and the introduction of Zn_SA_ and Pt_SA_ in Ti_2_CClx with STEM (Figure 4D–I).^[^ ^62^ ^]^ Figure 4J–O shows a HAADF‐STEM image of the Ni_SA_@N‐Ti_3_C_2_T_x_, in which the light dots are firmly distributed with a distance of ≈ 0.50 nm. Moreover, STEM energy dispersive X‐ray spectroscopy mapping shows the uniform distribution of Ti, C, Ni, and N elements.^[^ ^87^ ^]^ Although STEM is pivotal for identifying SACs, distinguishing SAs with similar atomic numbers can prove challenging. Hence, complementary characterization techniques are necessary to differentiate various SAs within materials. In addition, this technique can be extended to in situ STEM to trace the diffusion of SAs directly at different temperatures and specific reaction conditions.^[^ ^88^ ^]^

### 3.2 Synchrotron X‐Ray Absorption Spectroscopy

**Figure 5:**
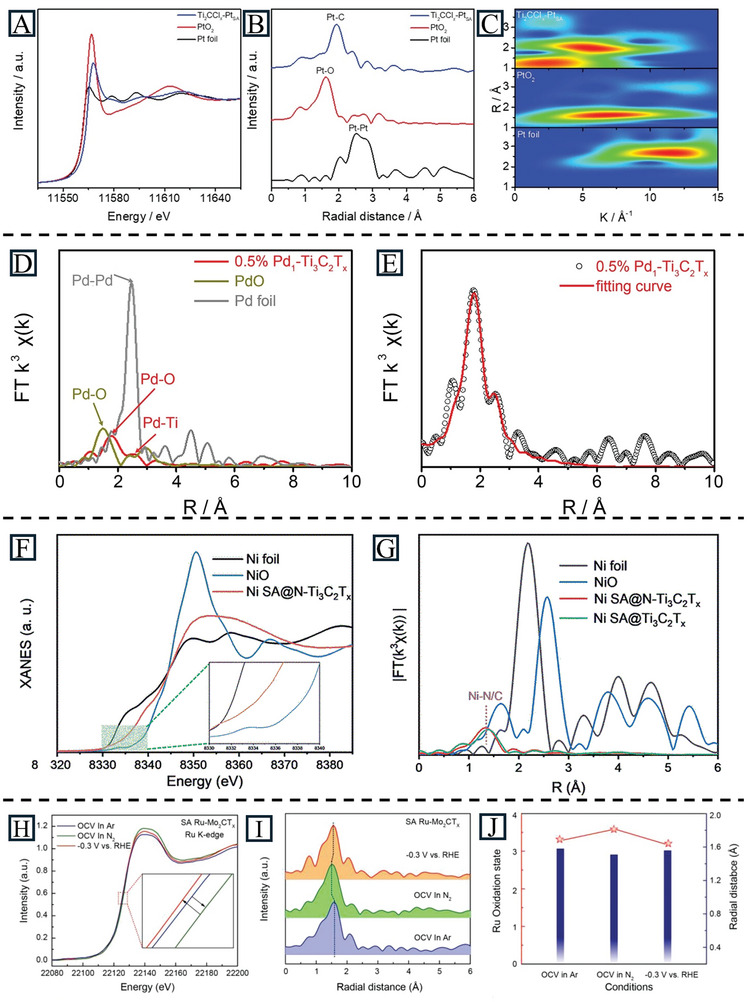
A) Pt L_3_‐edge XANES spectra, B) Fourier transforms of Pt L_3_, and C) WT Pt L_3_‐edge EXAFS spectra for Pt foil, PtO_2_, and Ti_2_CClx–Pt_SA_. Reproduced with permission.^[^ ^62^ ^]^ copyright 2024, Wiley‐VCH. D) Pd K‐edge EXAFS spectra of Pd foil, PdO, and 0.5% Pd_1_‐Ti_3_C_2_T_x_. E) EXAFS fitting curves for 0.5% Pd_1_‐Ti_3_C_2_T_x_. Reproduced with permission.^[^ ^85^ ^]^ Copyright 2024, Elsevier. The experimental Ni K‐edge F) XANES and G) EXAFS spectra of Ni SA@N‐Ti_3_C_2_T_x_ and counterparts. Reproduced with permission.^[^ ^87^ ^]^ Copyright 2024, The Royal Society of Chemistry. H) Normalized operando Ru K‐edge XANES spectra for SA Ru‐Mo_2_CT_X_ under various conditions (applied voltage versus RHE) in 0.5 M K_2_SO_4_ solution, insert is the magnified image. I) The corresponding FT‐EXAFS spectra derived from (H). J) The oxidation state of Ru and radial distance of the main peak under various conditions. Reproduced with permission.^[^ ^92^ ^]^ Copyright 2020, Wiley‐VCH.

XAS has outstanding advantages in studying the interactions between atoms in gas, liquid, and solid phases. It is widely used to determine the local structure and electronic structure which is hard to realize by traditional tools. According to the photon energy of X‐rays, XAS can be classified into soft XAS (<2 keV) and hard XAS (>4 keV).^[^ ^89^ ^]^ XAS spectra can be divided into two regions: X‐ray absorption near‐edge structure (XANES) and extended X‐ray absorption fine structure (EXAFS). XANES, within 30–50 eV of the absorption edge, is sensitive to the oxidation state and coordination chemistry.^[^ ^90^ ^]^ Thus, it can provide information on the atomic valance state with the position and shape of the absorption edge. EXAFS, in the range from 50 to 1000 eV or more, is caused by a single‐scattering or multi‐scattering process of photoelectrons with neighboring atoms. Therefore, it can provide information on bond length and the number of coordination species. Usually, these two forms of characterization can be assessed in the same scan. A simple single scan provides comprehensive detailed information, including oxidation states, bond lengths, bond angles, and the coordination number of active atoms. For SAs on MXenes, the dispersed SAs can be identified by the metal‐metal bonds in EXAFS spectra. XANES can further reveal the electronic and geometric coordination environment of SAs by comparison with reference samples. For example, Guo et al. employed EXAFS to analyze Pt SAs and Ti_2_CCl_x_. The Pt L_3_‐edge XANES spectra reveals that the absorption edge position of Ti_2_CCl_x_–Pt_SA_ lies between those of Pt and PtO_2_, suggesting a positive charge on the Pt SAs in Ti_2_CCl_x_–Pt_SA_ (**Figure** **5**A). From the Fourier transform (FT) of Pt L_3_‐edge EXAFS (Figure 5B), it showed the existence of Pt‐C (1.9 Å) instead of Pt‐Pt bonds (2.7 Å). The wavelet transform (WT) shows an intensity maxima of 3 Å^−1^ (Pt‐C) and 5 Å^−1^ (Pt‐Ti), implying that Pt is atomically dispersed in Ti_2_CCl_x_ (Figure 5C).^[^ ^62^ ^]^ Zhu et al. verified the atomic structure of Pd in Pd_1_‐Ti_3_C_2_T_x_ via Pd K‐edge EXAFS. The major peaks at 1.78 and 2.48 Å are attributed to Pd‐O and Pd‐Ti bonds, respectively, with no peaks for Pd‐Pd bonds (Figure 5D). By further fitting the EXAFS results, the coordination numbers of the Pd‐O and Pd‐Ti bonds were calculated to be 3.5 and 1.5, respectively. These results confirmed the presence of Pd SAs in the Pd_1_‐Ti_3_C_2_T_x_ catalyst (Figure 5E).^[^ ^85^ ^]^ Yang et al. studied the local coordination environment of Ni atoms in Ni_SA_@N‐Ti_3_C_2_T_x_. The adsorption edge position of Ni atoms, which is between Ni and NiO, indicates that the electronic structure of Ni^δ+^ (0 < δ < 2) is due to Ni‐support interactions (Figure 5F). With the FT‐EXAFS analysis, a peak at 1.45 Å proved that Ni atoms were connected to the substrate by Ni‐C/N bonds (Figure 5G).^[^ ^87^ ^]^

In addition to studying catalyst structure, operando XAS is also applied in the study of catalytic mechanisms, which provides the opportunity to monitor the behavior of active sites in MXene‐supported SACs at the atomic scale.^[^ ^91^ ^]^ For instance, Peng et al. studied the intrinsic catalytic activity of Ru_SA_@Mo_2_CT_x_ for the electrochemical nitrogen reduction reaction (NRR) with operando XAS. First, Ru_SA_@Mo_2_CT_x_ was tested in N_2_‐saturated and Ar‐saturated electrolytes respectively at open‐circuit potential (OCP). As shown in Figure 5H–J, this catalyst exhibited higher energy of the Ru K‐edge, increased Ru oxidation state, and shorter radial distance in N_2_‐saturated electrolytes. These changes may be attributable to the delocalization of unpaired electrons in the Ru 3d orbital, charge transfer from the Ru atom to the N atom, and the presence of Ru‐N bonds. Second, Ru_SA_@Mo_2_CT_x_ was tested at ‐0.3 V versus RHE in N_2_‐saturated electrolytes. When a potential was applied, the K‐edge shifted to a lower energy, the oxidation state decreased, and the radial distance (R) increased (Figure 5H–J). These results demonstrated the continuous reduction capability of Ru_SA_ centers for N_2_. The Ru SAs work highlights important roles in catalytic intermediate adsorption and electron back‐donation centers during the NRR process.^[^ ^92^ ^]^ While this technique shows many advantages, it is difficult to identify metal or metal oxide clusters in samples with SAs by XAS only, and other assisted characterization techniques are needed.

### 3.3 X‐Ray Photoelectron Spectroscopy

**Figure 6:**
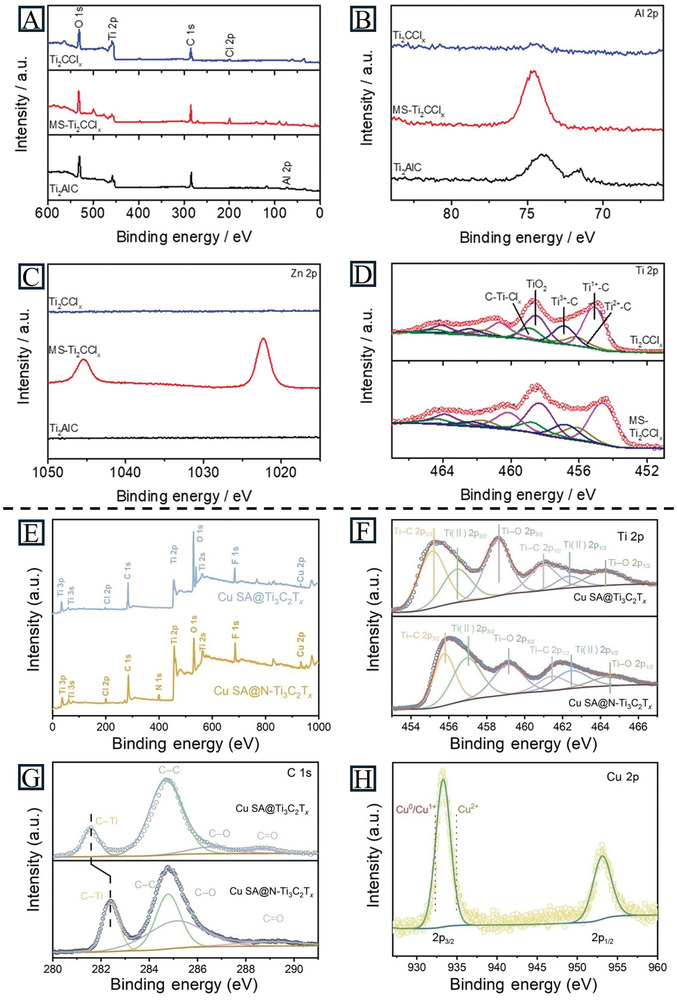
A) XPS survey spectra of Ti_2_AlC, MS‐Ti_2_CCl_x_, and Ti_2_CCl_x_. High‐resolution B) Al 2p, C) Zn 2p, and D) Ti 2p XPS spectra of Ti_2_AlC, MS‐Ti_2_CCl_x_, and Ti_2_CCl_x_. Reproduced with permission.^[^ ^62^ ^]^ Copyright 2024, Wiley‐VCH. E) XPS survey spectra of Cu_SA_@N‐Ti_3_C_2_T_x_ and Cu_SA_@Ti_3_C_2_T_x_, F) Ti 2p spectra, G) C 1s spectra, and H) Cu 2p spectra. Reproduced with permission.^[^ ^94^ ^]^ Copyright 2024, Springer Nature.

XPS, a highly surface‐sensitive method, can provide the composition and electronic states of elements on the surface of catalysts.^[^ ^93^ ^]^ For SACs, the SAs are dispersed in an unsaturated state and interact with the substrate, which is different from pure metals or metal oxides. By introducing suitable reactants and reaction temperatures to the XPS chamber, it can be upgraded to near‐ambient pressure XPS (NAP‐XPS), realizing the characterization of electrocatalysts under the reaction atmosphere. The element composition and surface states of Ti_2_CCl_x_ were characterized by XPS (**Figure** **6**A–D). Compared with the Ti_2_CCl_2_ prepared in molten salts (MS‐Ti_2_CCl_2_), there was no signal for Zn in Ti_2_CCl_2_ synthesized by ZnCl_2_ vapor, indicating the pure MXene with no metal impurities. Additionally, the peak shift of Ti in as‐prepared Ti_2_CCl_2_ revealed a higher oxidation state generated from Ti vacancies.^[^ ^62^ ^]^ Liu et al. characterized the successful preparation of Cu_SA_@N‐Ti_3_C_2_T_x_ with XPS (Figure 6E–H). The binding energy of C in Cu_SA_@N‐Ti_3_C_2_T_x_ was higher than that in Cu_SA_@Ti_3_C_2_T_x_, indicating the influence of N atoms doping. The Cu spectrum was deconvoluted into Cu^2+^ and Cu^0^ peaks, revealing a positive charge on the Cu atoms in Cu_SA_@N‐Ti_3_C_2_T_x_.^[^ ^94^ ^]^ However, the information obtained from XPS can only be used as supporting evidence and other techniques are needed to draw a definitive conclusion regarding the presence of SACs.

In addition, the in situ NAP‐XPS technique plays a crucial role in studying the behavior and thermal stability of atoms in catalysts. This enables the exploration of dynamic modification at a single atom‐support surface, guiding sophisticated defect design for future SACs. It can also be applied to Ti_3_C_2_T_x_ to detect the bonding environment and terminations.^[^ ^95^ ^]^

### 3.4 Raman Spectroscopy

**Figure 7:**
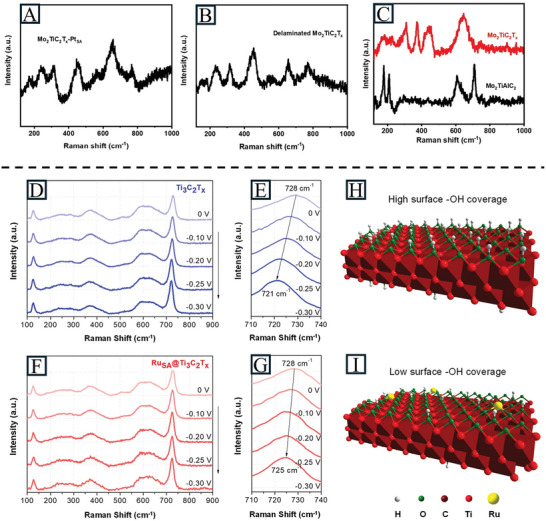
Raman spectra of A) Mo_2_TiC_2_T_x_‐Pt_SA_, B) delaminated Mo_2_TiC_2_T_x_, C) Mo_2_TiAlC_2_ and Mo_2_TiC_2_T_x_. Reproduced with permission.^[^ ^79^ ^]^ Copyright 2018, Springer Nature. D,E) In situ Spectro‐electrochemical Raman spectra of Ti_3_C_2_T_x_. F,G) In situ Raman spectra of Ru_SA_@Ti_3_C_2_T_x_. H,I) Schematic diagram of Ru_SA_@Ti_3_C_2_T_x_ surface under operando HER conditions. Reproduced under the terms of the CC‐BY 4.0 license.^[^ ^99^ ^]^ Copyright 2022, Wiley‐ VCH.

Raman spectroscopy (ex‐situ and operando) is a popular tool to obtain the structure of MXenes, where the A_1g_ and E_g_ vibrational modes represent out‐of‐plane and in‐plane vibrations, respectively.^[^ ^96^ ^]^ It also shows the presence of SAs on MXenes and identifies their anchoring sites by imaging modes. Moreover, it reveals the structure of SAs on MXenes by peak shift and changes in peak area ratio.^[^ ^97^ ^]^ In situ Raman spectroscopy is an especially powerful technique to trace the intermediates and the changes in the structure of MXene‐supported SACs under reaction conditions and identify the electrocatalytic centers.^[^ ^98^ ^]^ Zhang et al. confirmed the volumetric changes from Mo_2_TiAlC_2_ to Mo_2_TiC_2_T_x_ with peak changes in Raman spectra. The typical peaks at 178, 209, 607, and 708 cm^−1^ for Mo_2_TiAlC_2_ vanished, while strong peaks for Mo_2_TiC_2_T_x_ at 300–400 and 650 cm^−1^ were observed. The Raman spectra of Mo_2_TiC_2_T_x_‐Pt_SA_ and Mo_2_TiC_2_T_x_ delaminated by tetrabutylammonium hydroxide (TBAOH) showed the same peaks, demonstrating the successful exfoliation process (**Figure** **7**A–C).^[^ ^79^ ^]^ Zou et al. investigated time‐resolved information of Ru_SA_@Ti_3_C_2_T_x_ during the HER in alkaline electrolyte for mechanistic investigations with in situ Raman spectroscopy.^[^ ^99^ ^]^ The A_1g_ peak shift of Ru_SA_@Ti_3_C_2_T_x_ was smaller than that of Ti_3_C_2_T_x_, indicating a lower surface coverage of the ‐OH functional groups (Figure 7D–I). This showed that Ru SAs enhanced the adsorption of H, facilitating H_2_ generation at a lower potential and further reducing the protonation of exposed ‐O to ‐OH.

### 3.5 Theoretical Calculations

**Figure 8:**
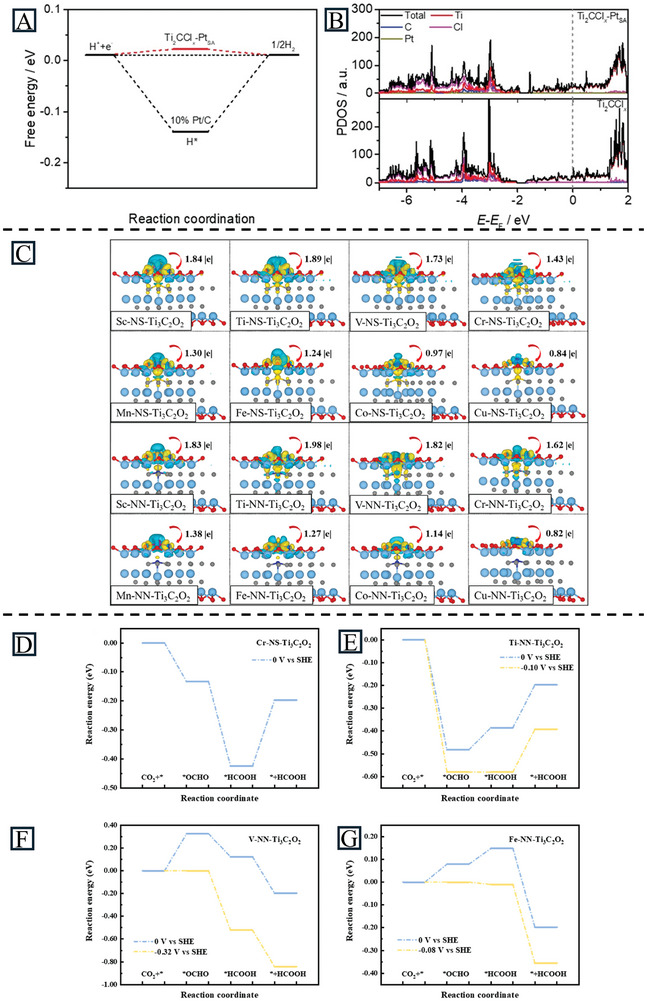
A) DFT‐calculated hydrogen adsorption Gibbs free energy of Ti_2_CCl_x_–Pt_SA_ and 10% Pt/C for HER. B) DOS of Ti_2_CCl_x_ and Ti_2_CCl_x_–Pt_SA_. Reproduced with permission.^[^ ^62^ ^]^ Copyright 2024, Wiley‐VCH. C) The charge density differences of TMs anchored onto NS/NN‐Ti_3_C_2_O_2_ supports. The charge depletion and accumulation are depicted by cyan and yellow, respectively. The isosurface value is 0.005 e/A3. The reaction mechanisms of ECO_2_RR to HCOOH under 0 V versus SHE and operando conditions on D) Cr‐NS‐Ti_3_C_2_O_2_, E) Ti‐NN‐Ti_3_C_2_O_2_, F) V‐NN‐Ti_3_C_2_O_2_, and G) Fe‐NN‐Ti_3_C_2_O_2_. Reproduced with permission.^[^ ^101^ ^]^ Copyright 2024, Elsevier Ltd.

In addition to the above characterizations, theoretical calculations also provide vital contributions to understanding SACs with atomistic precision, a feat sometimes challenging to attain through experimentation.^[^ ^100^ ^]^ First‐principles calculations, the most widely used method to simulate SACs, were employed to study the origin of outstanding HER performance for the Ti_2_CCl_x_‐Pt_SA_ catalyst. Based on first‐principles calculations, the hydrogen adsorption‐free energy of Ti_2_CCl_x_‐Pt_SA_ (0.016 eV) is closer to zero than that of 10% Pt/C (‐0.137 eV), meaning a more favorable hydrogen adsorption‐desorption process (**Figure** **8**A). The density of states (DOS) results, reflecting the electronic structure of materials, show that Ti_2_CCl_x_‐Pt_SA_ has a higher occupied state around the Fermi level than Ti_2_CCl_x_, resulting in good electrical conductivity and electrocatalytic performance (Figure 8B). This is consistent with the electrochemical results, where Ti_2_CCl_x_‐Pt_SA_ (E_onset_ = 19 mV, Tafel slope = 31 mV dec^−1^) shows superior HER activity than 10% Pt/C (E_onset_ = 29 mV, Tafel slope = 32 mV dec^−1^).^[^ ^62^ ^]^ Especially for MXene‐supported SACs, which are at the development stage, first‐principles calculations help to reveal their potential as electrocatalysts and give guidance for designing materials. Cao et al. investigated the coordination effect of MXene‐supported SACs for electrochemical carbon dioxide reduction reaction (ECO_2_RR) applications with DFT calculations. Based on the charge density difference of TM SAs on NS/NN‐Ti_3_C_2_O_2_ (Figure 8C), electrons are transferred from TMs to O atoms and N or S bonds, which can enhance the stability of TMs and optimize electronic structure to further improve ECO_2_RR performance. The reaction mechanism of four ideal ECO_2_RR catalysts toward HOOH was explored with reaction energy (Δ*G*). Negative Δ*G* means energetically favorable; positive Δ*G* means energy‐requiring; the step with the greatest Δ*G* is the potential determining step in the reaction process. Therefore, V/Fe‐NN‐Ti_3_C_2_O_2_, which consumes less energy, was predicted to exhibit superior catalytic activity with formation of *OCHO as the potential determining step (Figure 8D–G).^[^ ^101^ ^]^ Classical Molecular dynamics (MD) based on potential functions is a popular simulation method to model the formation of SAs.^[^ ^102^ ^]^ Cheng et al. examined the thermal stability of Zn/Mo_2_CO_2‐δ_ with MD and provided insights into its dynamic behavior at different temperatures. This study highlights the potential of Zn/Mo_2_CO_2‐δ_ for low‐temperature CO oxidation catalysis.^[^ ^103^ ^]^ However, precise predictions can only be made when the simulated model is close to real conditions. Instead of using typical first‐principles calculations based on the constant charge model, which cannot always accurately describe the electrochemical interface, the constant potential model has been developed to simulate real electrochemical systems. Ji et al. understand optimal Ru SAs site and expand the theoretical exploration under the constant potential model.^[^ ^104^ ^]^

In summary, various methods can be utilized to characterize the SAs within SACs, such as STEM, XAS, XPS, Raman, first‐principal calculations, and MD, among others. However, as each method presents its strengths and weaknesses, integrating multiple techniques is essential to arrive at a robust conclusion. Additionally, the characterizations and theoretical studies of MXene‐supported SACs based on these techniques are limited and need more effort to explore the accurate position of SAs, the relationship between the SAs and support materials, the behavior of SAs during electrochemical catalytic processes, and the possible promising MXene‐supported SACs for different applications. More in‐depth research could offer valuable guidance for future studies on MXene‐supported SACs.

## 4 Catalytic Applications of MXene‐Supported SACs

As 2D materials, MXenes possess remarkable properties that make them highly suitable for electrocatalytic applications. Their inherent metallic conductivity and hydrophilicity contribute to efficient charge transfer and favorable interactions with aqueous environments, respectively. Moreover, the unique MXene structure maximizes the exposure of active sites on the surface, further enhancing the catalytic performance. Thus, pristine MXene materials are promising alternatives for catalytic reactions. For instance, Ti_3_C_2_O_x_ shows a good HER performance compared with typical support materials with low overpotential (190 mV @ 10 mA cm^−2^) and low Tafel slope (60.7 mV dec^−1^).^[^ ^105^ ^]^ Single‐layer Ti_3_C_2_ exhibited a desirable ORR performance and stability in 0.1 M KOH by a four‐electron pathway.^[^ ^106^ ^]^ Parui et al. predicted Ti_2_C(OH)_2_ as a CO_2_RR catalyst with high reactivity and selectivity to yield formic acid.^[^ ^107^ ^]^ By introducing SAs onto the MXene structure, the catalytic efficiency can be significantly boosted. SA doping not only provides additional active sites for catalytic reactions but also allows precise control over the catalytic activity and selectivity. This level of control and enhancement often surpasses that achieved with conventional commercial catalysts, making MXene‐supported SACs highly promising for a wide range of electrocatalytic applications, including water splitting, CO_2_ reduction, and nitrogen reduction. Here, we delve into recent advancements in MXene‐supported SACs for electrocatalysis, encompassing studies on the HER, OER, ORR, CO_2_RR, and NRR, spanning both theoretical and experimental perspectives.

### 4.1 MXene‐Supported SACs for the HER

Hydrogen is considered one of the most promising candidates for the next generation of energy since it has a high energy density without carbon emissions.^[^ ^108^ ^]^ When it comes to hydrogen production, the HER via water splitting stands out as a cost‐effective and environmentally friendly approach.^[^ ^109^ ^]^ During this process, electrocatalysts play an important role and Pt is a near‐ideal HER electrocatalyst. However, scarcity and high cost limit its large‐scale usage in industrial applications. There are two strategies to overcome the challenges of Pt catalysts for the HER. One approach is to develop active and non‐noble alternatives to Pt such as transition metal sulfides, phosphates, carbides, and nitrides. MXenes possess high carrier mobilities and intrinsic layering, which benefit the HER process.^[^ ^110^ ^]^ However, the intrinsic electrocatalytic activity for reported MXenes is poor, where Mo_2_CT_x_ with high activity shows an overpotential of 283 mV at 10 mA cm^−2^ in a 0.5 M H_2_SO_4_.^[^ ^111^ ^]^ The other approach is to improve the active site utilization efficiency by limiting their size to clusters or even SAs. To maximize the benefits of these two strategies, many researchers try to disperse SAs on ideal support materials for high‐performance HER catalysts. The presence of heteroatoms on MXenes can provide new active sites and change the electronic structure, which affects the free energy of hydrogen adsorption and improves the performance of the HER process.^[^ ^112^ ^]^

**Figure 9:**
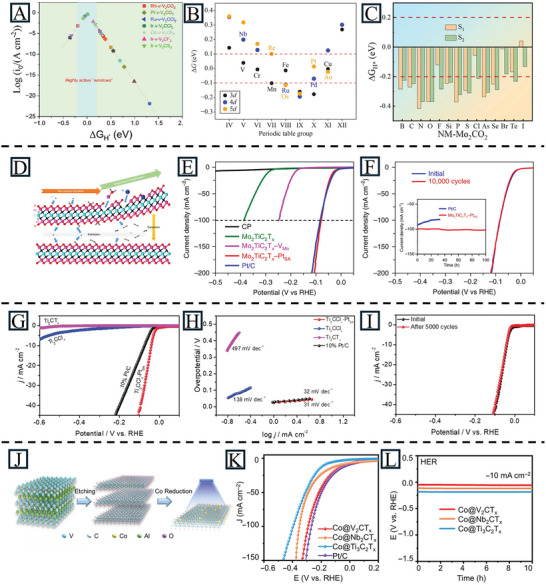
A) The calculated free‐energy diagrams for H adsorption on M−v−V_2_CT_2_ SACs. Reproduced with permission.^[^ ^113^ ^]^ Copyright 2023, Elsevier Inc. B) The variation in Δ*G* (H) with the Group number of substitutional impurities in the Nb_4_C_3_O_2_ monolayer. The points corresponding to Nb (representing the pristine Nb_4_C_3_O_2_) and impurities giving rise to the eV are labeled. Reproduced with permission.^[^ ^114^ ^]^ Copyright 2024, Springer Nature. C)The calculated ΔGH at the S1 and S2 sites on NM‐Mo_2_CO_2_ structures. Reproduced with permission.^[^ ^115^ ^]^ Copyright 2024, Elsevier B.V. D) Illustration of the synthesis mechanism for Mo_2_TiC_2_O_2_–Pt_SA_ during the HER process. E) HER polarization curves of carbon paper (CP), Mo_2_TiC_2_T_x_, Mo_2_TiC_2_T_x_–V_Mo_, Mo_2_TiC_2_T_x_–Pt_SA_ and Pt/C (40%), acquired using graphite rod as the counter electrode in 0.5 M H_2_SO_4_ solution. F) Stability test of Mo_2_TiC_2_T_x_–Pt_SA_ through potential cycling, before and after 10000 cycles. Inset: chronoamperometry curve of Mo_2_TiC_2_T_x_–Pt_SA_ and Pt/C. Reproduced with permission.^[^ ^79^ ^]^ Copyright 2018, Springer Nature. G) HER polarization curves of Ti_2_CT_x_, Ti_2_CClx, Ti_2_CCl_x_–Pt_SA_, and 10% Pt/C collected in 0.5 M H_2_SO_4_ electrolyte. H) Tafel plots of Ti_2_CT_x_, Ti_2_CClx, Ti_2_CCl_x_–Pt_SA_, and 10% Pt/C. I) The stability of Ti_2_CCl_x_–Pt_SA_. Reproduced with permission.^[^ ^62^ ^]^ Copyright 2024, Wiley‐VCH. J) Schematic illustration of the fabrication of the Co@MXene composites on an example of V_2_CT_x_; K) HER LSV curves of Co@V_2_CT_x_, Co@Nb_2_CT_x_, Co@Ti_3_C_2_T_x_ and Pt/C electrodes at 5 mV s^−1^; L) HER chronopotentiometry response of three listed electrodes at a current density of 10 mA cm^−2^. Reproduced under the terms of the CC‐BY 4.0 license.^[^ ^117^ ^]^ Copyright 2022, Wiley‐VCH GmbH.

To guide the design of MXene‐supported SACs for the HER, many researchers employ theoretical analyses. According to DFT studies, many MXene‐supported SACs exhibit potential for HER activities in acidic and alkaline electrolytes, such as Ir/Ru/Pt‐v‐V_2_CCl_2_,^[^ ^113^ ^]^ Fe/Cr/Cu@Nb_4_C_3_O_2_,^[^ ^114^ ^]^ and Cl/Br/I‐Mo_2_CO_2_^[^ ^115^ ^]^ etc. Wang et al. tuned the coordination microenvironment of V_2_CT_x_‐based SACs (T = O, F, S, and Cl) by DFT calculations. They also showed that different surface groups play important roles in catalytic activity. Especially, the small ΔG_H_ of Ir‐v‐V_2_CCl_2_ (0.01 eV), Ru‐v‐V_2_CCl_2_‐(0.04 eV), and Pt‐v‐V_2_CO_2_ (−0.08 eV) are even better than commercial Pt (−0.09 eV) (**Figure** **9**A).^[^ ^113^ ^]^ Šljivančanin calculated the free energy of H adsorption to design O‐terminated SACs. By introducing TM and noble metal atoms, the free energy approached that of Pt. They also investigated the relationship between charge transfer and local reactivity (Figure 9B).^[^ ^114^ ^]^ Besides metal atoms, non‐metal SAs are also used as active sites to improve the HER performance of MXene materials. Zhang et al. studied the impacts of single non‐metal atom (Cl, Br, and I) doping on the HER activity of molybdenum carbide MXene using DFT calculations. With the help of Cl, Br, and I, the ΔG_H_ is close to 0 eV, meaning superior HER performance (Figure 9C).^[^ ^115^ ^]^ These theoretical results provide valuable insights into the construction and mechanism of MXene‐supported SACs for the HER.

Some MXene‐supported SACs have been shown experimentally to possess superior HER performance. Various noble metal atoms have been successfully introduced into MXenes. For example, Pt_SA_ was introduced into Mo_2_TiC_2_T_x_, Ti_3_C_2_T_x_/SWCNTs, and Ti_3_C_2_T_x_, showing improved HER activities. In Mo_2_TiC_2_T_x_–Pt_SA_, the surface Mo vacancies work as anchoring sites for the SAs and the Pt atoms can be further stabilized by forming strong covalent bonds (Pt‐C). A low overpotential of −30 mV at 10 mA cm^−2^ was achieved with outstanding stability for 100 h with active sites of Mo‐O and Pt, resulting in a 39.5‐times increase in mass activity compared to commercial Pt/C (Figure 9D–F).^[^ ^79^ ^]^ A self‐supported Ti_3_C_2_T_x_@Pt/SWCNTs catalyst was also prepared, showing an overpotential of −62 mV at 10 mA cm^−2^ and remaining stable for 800 h.^[^ ^116^ ^]^ Guo et al. also employed vacancies to design a Ti_2_CT_x_‐Pt_SA_ catalyst that fixed Pt SAs on the Ti vacancies, achieving an overpotential of −41 mV at 10 mA cm^−2^ and good stability over 5000 LSV cycles (Figure 9G–I).^[^ ^62^ ^]^ Ramalingam et al. doped Ru SAs on N‐S‐Ti_3_C_2_T_x_ through the formation of Ru‐N and Ru‐S bonds. Due to the high electronegativity and different atomic radii of N and S atoms, they served as binding sites for Ru SAs. The catalyst possessed a small ΔG_H_ of 0.08 eV and showed good HER activity under various pH conditions (76 mV at 10 mA cm^−2^ in acidic electrolyte; 275 mV at 10 mA cm^−2^ in neutral electrolyte; 99 mV at 10 mA cm^−2^ in alkaline electrolyte).^[^ ^84^ ^]^ Pd SAs are also successfully doped into Ti_3_C_2_T_x_ with regulated reaction kinetics. The as‐prepared SAC, 0.5% Pd_1_‐Ti_3_C_2_T_x_, possessed an overpotential of 154 mV at 10 mA cm^−2^ and a Tafel slope of 70 mV dec^−1^, much better than Ti_3_C_2_T_x_ and Pd nanoparticles in Ti_3_C_2_T_x_.^[^ ^85^ ^]^ In addition to noble metal atoms, non‐noble metals have also been introduced into MXenes, achieving efficiencies comparable to noble metal‐doped MXenes. Zhao et al. anchored Co atoms onto MXenes (Nb_2_CT_x_, V_2_CT_x_, and Ti_3_C_2_T_x_) to enhance the HER properties. Co@V_2_CT_x_ possessed an outstanding HER overpotential of −35 mV and good stability for 10 h in 1 M KOH (Figure 9J–L).^[^ ^117^ ^]^ Zhao et al. constructed a dual‐atom catalyst CoNi‐Ti_3_C_2_T_x_ by a surface modification method, where the Co and Ni were fixed in Ti_3_C_2_T_x_ via metal‐O or metal‐N bonds. It displayed an HER overpotential of −31 mV at 10 mA cm^−2^ with only 6.3% degradation after 100 h at a current density of 500 mA cm^−2^.^[^ ^118^ ^]^ Based on the data presented in **Table** **2**, MXene‐supported SACs exhibit a favorable HER performance when compared to other 2D‐supported SACs.

**Table 2:** Summary of electrocatalytic performance of MXenes, MXene‐supported SACs, and other 2D‐supported SACs.

HER catalysts	Research type	Substrate	Electrolyte	Overpotential η at 10 mA cm^−2^ [mV]	Tafel slope [mV dec^−1^]	Stability [hour/cycle]	Loading amount [wt.%]	Refs.
Mo_2_CT_x_(T = O, OH)	E	GC	0.5 M H_2_SO_4_	283	70.0	2 h	/	[111]
E‐Ti_3_C_2_T_x_(F, OH, O)	E	GC	0.5 M H_2_SO_4_	266	109.8	/	/	[105]
E‐Ti_3_C_2_O_x_	E	GC	0.5 M H_2_SO_4_	190	60.7	2000	/	[105]
E‐Ti_3_C_2_(OH)_x_	E	GC	0.5 M H_2_SO_4_	217	88.5	/	/	[105]
Ti_2_CCl_x_‐Pt _SA_	E	GC	0.5 M H_2_SO_4_	41	31.0	18 h/5000	0.94	[62]
Mo_2_TiC_2_T_x_–Pt_SA_	E	CFP	0.5 M H_2_SO_4_	30	30.0	‐/10 000	1.20	[79]
Ru_SA_‐N‐S‐Ti_3_C_2_T_x_	E	CFP	0.5 M H_2_SO_4_ 0.5 M Na_2_SO_4_ 0.5 M NaOH	76 275 99	90.0 / /	16 h/‐ 1000 4000	1.20	[84]
Pd _SA_‐Ti_3_C_2_T_x_	E	NF	1 M KOH	154	70.0	40h	0.5	[85]
Co _SA_@V_2_CT_x_	E	CC	1 M KOH	35	109.1	10h	1.08	[117]
CoNi‐Ti_3_C_2_T_x_	E	CC	1 M KOH	31	33.0	100h	5.60	[118]
Co_SA_‐N‐graphene	E	Graphene paper	0.5 M H_2_SO_4_ 1 M NaOH	147 270	82.0 /	10 h/1000 10 h/1000	0.57	[136]
Co/Se‐MoS_2_‐NF	E	NF	0.5 M H_2_SO_4_	104	29.0	360 h	10.40	[137]
20% Pt/C (Commercial)	E	CFP CFP	0.5 M H_2_SO_4_ 1 M KOH	13 28	32.0 24.0	/ /	20.00 20.00	[80, 112]
Ti_3_C_2_I_2_‐Ir	T	/	/	|ΔGH|<0.09eV	/	stable	/	[40]
Ti_3_C_2_Br_2_‐Cu	T	/	/	|ΔGH|<0.09eV	/	stable	/	[40]
Ti_3_C_2_Br_2_‐Pt	T	/	/	|ΔGH|<0.09eV	/	stable	/	[40]
Ti_3_C_2_Cl_2_‐Cu	T	/	/	|ΔGH|<0.09eV	/	stable	/	[40]
Ti_3_C_2_Cl_2_‐Pt	T	/	/	|ΔGH|<0.09eV	/	stable	/	[40]
Ti_3_C_2_Se_2_‐Au	T	/	/	|ΔGH|<0.09eV		stable	/	[40]
Ti_3_C_2_Te_2_‐Nb	T	/	/	|ΔGH|<0.09eV		stable	/	[40]
Ir‐v‐V_2_CCl_2_	T	/	/	|ΔGH| = 0.01eV		stable	/	[113]
Ru‐v‐V_2_CCl_2_	T	/	/	|ΔGH| = 0.04eV		stable	/	[113]
Pt‐v‐V_2_CCl_2_	T	/	/	|ΔGH| = 0.08eV		stable	/	[113]
Pt(111)	T	/	/	|ΔGH| = 0.09eV		stable	/	[40]

OER catalysts	Research Type	Substrate	Electrolyte	Overpotential η at 10 mA cm^−2^ [mV]	Tafel slope [mV dec^−1^]	Stability [hour/cycle]	Loading amount [wt.%]	Refs.
Co _SA_@V_2_CT_x_	E	CC	1 M KOH	245	90.4	10 h	1.58	[117]
CoNi‐Ti_3_C_2_T_x_	E	CC	1 M KOH	298	79.8	100 h	5.60	[118]
Ni‐O‐G SACs	E	CC	1 M KOH	224	42.0	50 h	3.10	[138]
Ni‐graphene	E	GC	1 M KOH	301	63.0	2000cycle	0.23	[139]
Pt SA‐Fe‐N‐C	E	GC	0.1 M KOH	310	62.0	50 h	2.10	[140]
Rh‐v‐V_2_CS_2_	T	/	/	300	/	/	/	[113]
Pt‐v‐V_2_CO_2_	T	/	/	360	/	/	/	[113]
Rh‐v‐V_2_CO_2_	T	/	/	370	/	/	/	[113]
Rh‐v‐V_2_CCl_2_	T	/	/	490	/	/	/	[113]
Pt‐v‐V_2_CCl_2_	T	/	/	490	/	/	/	[113]
IrO_2_	T	/	/	650	/	/	/	[113]

ORR catalysts	Research Type	Substrate	Electrolyte	Overpotential η at 10 mA cm^−2^ [mV]	Tafel slope [mV dec^−1^]	Stability [hour/cycle]	Loading amount [wt.%]	Refs.
Pt‐v‐V_2_CO_2_	T	/	/	380	/	/	/	[113]
Rh‐v‐V_2_CS_2_	T	/	/	400	/	/	/	[113]
Pt‐v‐V_2_CCl_2_	T	/	/	550	/	/	/	[113]
Pd/Ti_3_C_2_S_2_	T	/	/	<500	/	/	/	[123]
Pd/Ti_3_C_2_O_2_	T	/	/	<500	/	/	/	[123]

CO_2_RR catalysts	Research Type	Substrate	Electrolyte	Product	Selectivity	Faradaic Efficiency	Loading amount [wt.%]	Refs.
Cu_SA_‐Ti_3_C_2_T_x_ (T = O, OH,F)	E	CC	1 M KOH	EtOH/C2H4	98	71.0%	0.20	[128]
Cu_SA_@N‐Ti_3_C_2_T_x_(T = O, OH,F)	E	GC	0.5 M KHCO_3_	CO	>97%	97.4%	/	[94]
Cu_SA_@Ti_3_C_2_T_x_	E	GC	0.5 M KHCO_3_		>66%	/	/	[94]
Cu_SA_‐Ti_3_C_2_Cl_x_	E	GC	0.1 M KHCO_3_	CH3OH	/	59.1%	1.00	[141]
Ni_SA_‐graphene	E	GC	0.5 M KHCO_3_	CO	97%	95.0%	0.44 at. %	[142]
Mn_SA_‐C_3_N_4_/CNT	E	CC	0.5 M KHCO_3_	CO	/	98.8%	0.17	[143]
Vo‐CuO(Sn)	E	CC	0.1 M KHCO_3_	CO	95%	99.9	3.15 at. %	[144]
Co‐Mo_2_CS_2_	T	/	/	CH_4_	−0.304 (*U_L_*)	/	/	[145]
Ni_SA_‐Mo_2_CS_2_	T	/	/	CHOOH	−0.269 (*U_L_*)	/	/	[145]
Fe_SA_‐Mo_2_CS_2_	T	/	/	CO	−0.245 (*U_L_*)	/	/	[145]
Cr_SA_‐Mo_2_CS_2_	T	/	/	CH_4_	−0.296 (*U_L_*)	/	/	[145]
Cr_SA_@Mo_2_CS_2_	T	/	/	CH_4_	−0.450 (*U_L_*)	/	/	[127]
Ni_SA_‐Ov‐Ti_2_CO_2_	T	/	/	HCOOH	−0.27 (*U_L_*)	/	/	[146]
Co‐NS‐Ti_3_C_2_O_2_	T	/	/	HCOOH	−0.23 (*U_L_*)	/	/	[101]
Ti‐NN‐Ti_3_C_2_O_2_	T	/	/	HCOOH	−0.19 (*U_L_*)	/	/	[101]
V‐NN‐Ti_3_C_2_O_2_	T	/	/	HCOOH	−0.32 (*U_L_*)	/	/	[101]
Fe‐NN‐Ti_3_C_2_O_2_	T	/	/	HCOOH	−0.08 (*U_L_*)	/	/	[101]

NRR catalysts	Research Type	Substrate	Electrolyte	Product	Faradaic Efficiency		Loading amount [wt.%]	Refs.
Fe_SA_/Ti_3_C_2_T_x_	E	/	0.1 M Na_2_SO_4_	NH_3_	82.90%		1.69	[135]
RuSA‐Mo_2_CT	E	CFP	0.5 M K_2_SO_3_	NH_3_	25.77%		1.41	[92]
RuSA@rGO/NC	E	/	HCl	NH_3_	17.90%		0.95	[147]
Fe‐MoS _2_	E	CFP	0.5 M K_2_SO_3_	NH_3_	18.80%		5.3 at. %	[148]
Au_1_/C_3_N_4_	E	CFP	0.05 M H_2_SO_4_	NH_3_	11.10%		0.15	[149]
Ag/Ov‐MXene	T	/	/	NH_3_	−0.24 (*U_L_*)		/	[132]
Cu/Ov‐MXene	T	/	/	NH_3_	−0.34 (*U_L_*)		/	[132]
V1@Ti_2_CO_2_	T	/	/	NH_3_	−0.17 (*U_L_*)		/	[150]

Abbreviations: E, experiment; T, theoretical; GC, glass carbon; CFP, carbon fiber paper; NF, nickel foam, GP, graphene paper, *U_L_*, limiting potential.

### 4.2 MXene‐Supported SACs for the OER and ORR

In addition to the HER, the OER and ORR are also indispensable for developing clean H_2_. The OER, the anodic half‐reaction in water splitting, affects the potential required to drive the overall water‐splitting reaction.^[^ ^8c^ ^]^ The ORR plays an important role in proton exchange membrane (PEM) fuel cells which helps to generate electricity from clean H_2_.^[^ ^119^ ^]^ The sluggish kinetics of the OER and ORR are further limitations to developing clean energy, and it is essential to develop novel and efficient ORR/OER catalysts to reduce the high overpotentials.^[^ ^120^ ^]^ Currently, noble metals (e.g., Ru and Ir), metal oxides, and metal sulfides are common OER catalysts.^[^ ^121^ ^]^ Although these advanced electrocatalysts can achieve outstanding OER performances, they tend to experience elemental leaching and particle agglomeration under high voltage conditions, which affects catalyst stability. As such, 2D MXenes are emerging as a new candidate for OER catalyst support due to their unique properties.

**Figure 10:**
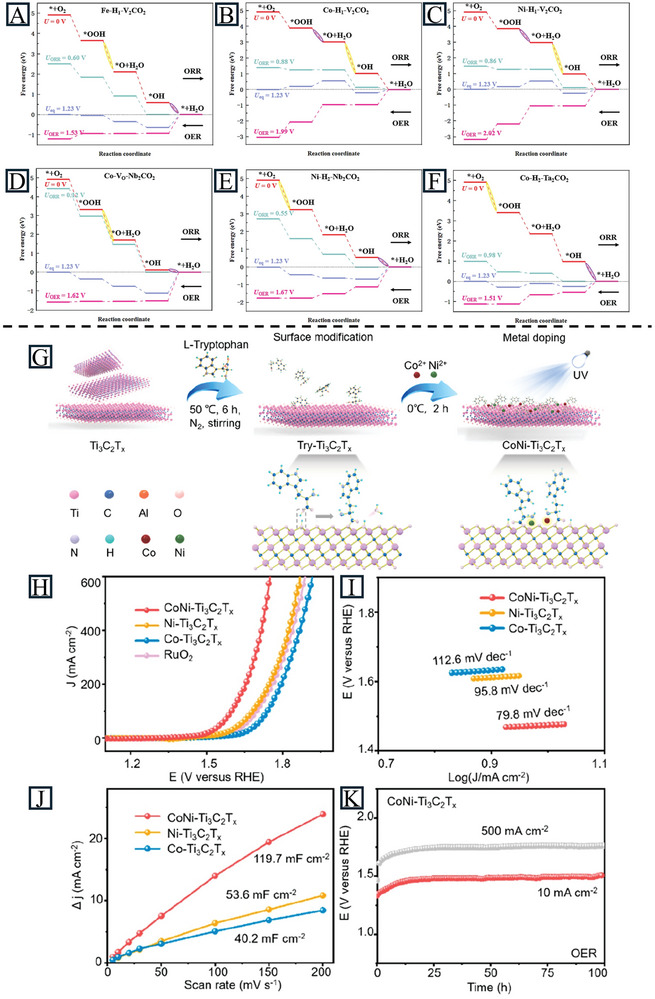
Free energy diagrams of A) Fe‐H_1_‐V_2_CO_2_, B) Co‐H_1_‐V_2_CO_2_, C) Ni‐H_1_‐V_2_CO_2_, D) Co‐V_O_‐Nb_2_CO_2_, E) Ni‐H_2_‐Nb_2_CO_2_, and F) Co‐H_2_‐Ta_2_CO_2_. The steps marked in purple and yellow represent the PDS of the ORR and OER, respectively. Reproduced with permission.^[^ ^124^ ^]^ Copyright 2024, Elsevier Inc. G) Schematic illustration of anchoring l‐tryptophan at the surface of Ti_3_C_2_T_x_ MXene, followed by fabrication of dual‐atom CoNi‐Ti_3_C_2_T_x_ composites. H) OER LSV curves of CoNi‐Ti_3_C_2_T_x_, Ni‐Ti_3_C_2_T_x_, Co‐Ti_3_C_2_T_x_, and RuO_2_ electrodes at 5 mV s^−1^. I) Tafel slopes of CoNi‐Ti_3_C_2_T_x_, Ni‐Ti_3_C_2_T_x_, and Co‐Ti_3_C_2_T_x_ electrodes. J) C_dl_ values of CoNi‐Ti_3_C_2_T_x_, Ni‐Ti_3_C_2_T_x_ and Co‐Ti_3_C_2_T_x_. K) OER chronopotentiometry response of CoNi‐Ti_3_C_2_T_x_ electrodes at current densities of 10 and 500 mA cm^−2^. Reproduced with permission.^[^ ^118^ ^]^ Copyright 2024, American Chemical Society.

Ram et al. investigated the OER performance of 21 individual TM atoms on Mo_2_CO_2_ by DFT calculations. TM atoms act as OER active sites and can exchange charge with the Mo_2_CO_2_ MXene.^[^ ^122^ ^]^ Cao et al. designed a series of TM atoms supported on Ti_3_C_2_S_2_, analyzed by DFT calculations. Pd/Ti_3_C_2_S_2_ and Ir/Ti_3_C_2_S_2_ were found to be promising ORR catalysts with an overpotential lower than 0.5 V. SAs fixed on Ti_3_C_2_S_2_ exhibited better catalytic activity than Ti_3_C_2_O_2_.^[^ ^123^ ^]^ In addition to these widely used MXenes, Dai et al. constructed efficient bifunctional catalysts for the ORR and OER by theoretical calculations with V_2_CO_2_ and Nb_2_CO_2_.^[^ ^124^ ^]^ All of these results provide theoretical support and guidance for designing ORR and OER electrocatalysts with MXene materials. As shown in **Figure** **10**A–F, the Gibbs free energy diagrams of catalysts are studied to explore the ORR/OER thermodynamics. The potential‐determining step in the ORR process of Co‐H_1_‐V_2_CO_2_ and Ni‐H_1_‐V_2_CO_2_ is the second proton‐coupled electron transfer step, while that of Fe‐H_1_‐V_2_CO_2_, Co‐Vo‐Nb_2_CO_2_, Ni‐H_2_‐Nb_2_CO_2,_ and Co‐H_2_‐Ta_2_CO_2_ is the fourth proton‐coupled electron transfer step. For the OER process, Fe‐H_1_‐V_2_CO_2_ and Co‐Vo‐Nb_2_CO_2_ are limited by the *O to *OOH step, while Co‐H_1_‐V_2_CO_2_ and Ni‐H_1_‐V_2_CO_2_ are limited by the *OH to *O step. Based on their model, Co‐H_2_‐Ta_2_CO_2_ shows the lowest potential gap value (0.53 V), meaning a good overpotential for bifunctional catalytic performance.

Some of the MXene‐supported SACs have experimentally demonstrated good ORR/OER performance. Zhao et al. further studied the electronic structure and electrocatalytic activity by synthesis of Co SAs on MXenes (V_2_CT_x_, Nb_2_CT_x_, and Ti_3_C_2_T_x_) via a photochemical reduction method. Interestingly, Co@V_2_CT_x_ not only exhibited a promising OER performance (242 mV at 10 mA cm^−2^) but also possessed remarkable HER activity (35 mV at 10 mA cm^−2^). This is due to the high electron transfer in Co@V_2_CT_x_, which redistributes the electronic structure of Co and lowers the energy barriers in the HER and OER processes.^[^ ^117^ ^]^ They also extended it to dual‐atom Co/Ni catalysts doped on MXenes (Figure 10G–K). The synthesized CoNi‐Ti_3_C_2_T_x_ displayed an OER overpotential of 241 mV and high stability for 100 h at an industrially relevant current density (500 mA cm^−2^).^[^ ^118^ ^]^ As indicated in Table 2, MXenes demonstrate a notable ability to accommodate a higher density of SAs, thereby, enhancing the OER performance in alkaline electrolytes.

### 4.3 MXene‐Supported SACs for the CO_2_RR and CORR

Significant CO_2_ emissions from burning fossil fuels have contributed greatly to global climate change. Besides considering alternative clean energy sources, many researchers focus on capturing and transforming CO_2_ into valuable resources to address this issue. Among them, the CO_2_ electrocatalytic reduction reaction can realize the direct conversion of CO_2_ into carbon fuels, such as CO, HCOOH, CH_4_, C_2_H_4_, C_2_H_6,_ and C_2_H_5_OH.^[^ ^125^ ^]^ However, several challenges need to be addressed to facilitate the CO_2_RR, such as high overpotential, low selectivity for the target product, poor CO_2_ solubility, and low efficiency due to the HER.^[^ ^126^ ^]^ Therefore, a cost‐effective and efficient catalyst is required to enhance CO_2_ conversion efficiency. MXenes have attracted attention for CO_2_RR application due to the improved chemical activity and selectivity, which show the potential to overcome the limitations of traditional CO_2_RR catalysts.

**Figure 11:**
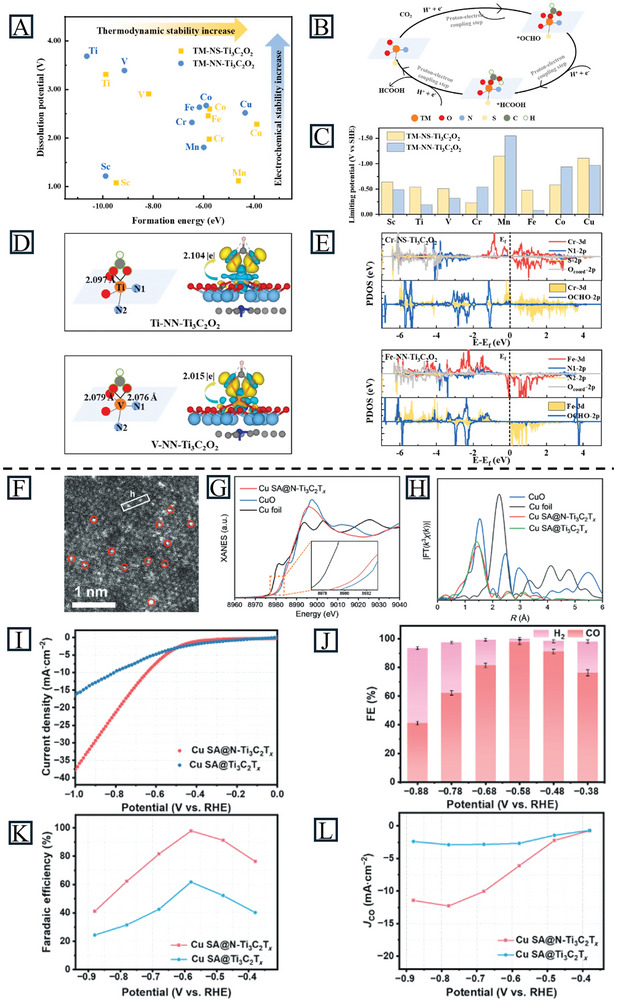
A) The computed formation energy and dissolution potential of TMs in TM‐NS/NN‐Ti_3_C_2_O_2_. B) Schematic illustration of the reaction mechanism of ECO_2_RR toward HCOOH on TM‐NS/NN‐Ti_3_C_2_O_2_. C) Theoretical limiting potential *U_L_* of ECO_2_RR toward HCOOH on TM‐NS/NN‐Ti_3_C_2_O_2_. D) Adsorption configurations of *OCHO intermediate and charge density difference on Ti‐NN‐Ti_3_C_2_O_2_ and V‐NN‐Ti_3_C_2_O_2_. The isosurface values are 3 × 10^−3^ e Bohr^−3^. Yellow and cyan regions represent increasing and decreasing electron densities, respectively. E) Projected density of states of catalysts and *OCHO adsorption on catalysts of Cr‐NS‐Ti_3_C_2_O_2_ and Fe‐NN‐Ti_3_C_2_O_2_. Reproduced with permission.^[^ ^101^ ^]^ Copyright 2024, Elsevier Ltd. F) HAADF‐STEM image of Cu_SA_@N‐Ti_3_C_2_T_x_. The experimental Cu K‐edge G) XANES and H) EXAFS spectra of Cu_SA_@N‐Ti_3_C_2_T_x_ and counterparts. I) LSV curves of Cu_SA_@N‐Ti_3_C_2_T_x_, performed in CO_2_‐saturated 0.5 M KHCO_3_. J) Potential‐dependent FEs of H_2_ and CO for Cu_SA_@N‐Ti_3_C_2_T_x_, at different potentials. K) The CO FEs of Cu_SA_@N‐Ti_3_C_2_T_x_ and Cu_SA_@Ti_3_C_2_T_x_ at different potentials. L) Jco of Cu_SA_@N‐Ti_3_C_2_T_x_ and Cu_SA_@Ti_3_C_2_T_x_. Reproduced with permission.^[^ ^94^ ^]^ Copyright 2024, Springer Nature.

DFT calculations are commonly used to design MXene‐supported SACs for the ECO_2_RR. Li et al. analyzed SACs of TM@Ti_2_CT_x_ (T = ‐O, ‐S) monolayers for the CO_2_RR by first‐principles calculations. TM@Ti_2_CO_2_ could better activate CO_2_ molecules than TM@Ti_2_CS_2_, while the theoretical limiting potential (*U*_L_)of the potential determining step of TM@Ti_2_CS_2_ is smaller than TM@Ti_2_CO_2_. This study offers new insights into designing catalysts by combining the advantages of different functional groups.^[^ ^127^ ^]^ Cao et al. employed DFT to explore the ECO_2_RR activity toward HCOOH on two types of MXene‐supported SACs, TM‐NS‐Ti_3_C_2_O_2_ and TM‐NN‐Ti_3_C_2_O_2_, where TM was Sc, V, Ti, Mn, Cr, Cu, Fe, Co, and Ni.^[^ ^101^ ^]^ For TM‐NS‐Ti_3_C_2_O_2_, TM was coordinated with two O atoms, one S atom, and one N atom, while it was coordinated with two O atoms and two S atoms in TM‐NN‐Ti_3_C_2_O_2_. A catalyst with good stability should have a formation energy below 0 and a dissolution potential above 0, where all the TM‐NN/NS‐Ti_3_C_2_O_2_ (TMs in the first row) meet these stability criteria (**Figure** **11**A). During the reaction process, the adsorbed CO_2_ on the catalysts’ surface led to the formation of *OCHO. The dangling O atoms were then attacked by protons to form *HCOOH, which would be finally desorbed as HCOOH (Figure 11B). Based on the above mechanism, the reaction energies of reactions on all catalysts were calculated and employed *U*_L_ as a descriptor. According to the calculated *U*_L_ values, Cr‐NS‐Ti_3_C_2_O_2_ (−0.23 V versus SHE) and Ti/V/Fe‐NN‐Ti_3_C_2_O_2_ (−0.19, −0.32, and −0.08 V versus SHE) whose *U*_L_ values are more positive than that of a benchmark SnO_2_ catalyst (−0.37 V versus SHE), are good choices for HCOOH production (Figure 11C). The adsorption and hydrogenation of the *OCHO intermediate is strongly associated with the catalytic activity. Further electronic structure analyses of *OCHO adsorption on catalysts revealed that both the electronic interaction mechanism and spin polarization nature of SACs affect the *OCHO intermediate. More electron transfer in Ti/V‐NN‐Ti_3_C_2_O_2_ and large spin polarization of Cr and Fe in Cr‐NS‐Ti_3_C_2_O_2_ and Fe‐NN‐Ti_3_C_2_O_2_ are responsible for their outstanding ECO_2_RR performance toward HCOOH (Figure 11D,E).

Bao et al. synthesized Cu_SA_/Ti_3_C_2_T_x_ where the Cu SAs corresponded to O‐coordinated Cu sites on the MXene matrix as carbon monoxide reduction reaction (CORR) catalysts.^[^ ^128^ ^]^ In 1 M KOH saturated with CO, the catalyst achieved a total Faradaic efficiency (FE) of 98% at −0.7 V versus RHE, with product selectivity of over 79% toward C_2_ products (EtOH and C_2_H_4_) at −0.6 to −0.9 V versus RHE. Compared to Cu_NP_/Ti_3_C_2_T_x_, Cu_SA_/Ti_3_C_2_T_x_ exhibited a 3.2‐fold increase in current density at −1.0 V versus RHE (−52.2 mA cm^−2^ vs. −16.2 mA cm^−2^). It also showed a significant improvement over Cu_NP_/Ti_3_C_2_T_x_ in the FE of C_2_H_4_ and EtOH (21% at −0.7 V versus RHE). According to theoretical calculations, Cu SAs coordinated with oxygen remain stable during the reduction reaction, promoting the formation of intermediates and decreasing the free energy barrier of the rate‐determining step. The good reactivity, structural simplicity, and uniform catalytic sites of Cu_SA_/Ti_3_C_2_T_x_ contribute to the good selectivity of C_2_ products. Liu et al. incorporated Cu SAs into N‐doped Ti_3_C_2_T_x_ for the CO_2_RR. The HAADF‐STEM images (Figure 11F) and XAS spectra (Figure 11G,H) demonstrate the existence of Cu SAs.^[^ ^94^ ^]^ Cu_SA_@N‐Ti_3_C_2_T_x_ exhibited a lower overpotential than Cu_SA_@Ti_3_C_2_T_x_ and more efficient CO_2_ conversion (Figure 11I). With the help of C‐Cu‐N bridge fragments, the CO FE reached 97.4% at −0.58 V (Figure 11J), improving the CO FE (Figure 11K) and J_CO_ (Figure 11L) of Cu_SA_@Ti_3_C_2_T_x_ remarkably.

Hence, the catalytic performance of MXenes for the CO_2_RR is influenced by the functional groups, SAs, and the type of metal (M) present. The current MXene‐support SACs for CO_2_RR are summarized in Table 2. However, there is limited experimental research in this area, necessitating further in‐depth investigation in the future.

### 4.4 MXene‐Supported SACs for the NRR and NO_3_RR

NH_3_, a vital chemical feedstock, is essential in industrial and agricultural production, as well as energy storage and conversion which could transport and supply hydrogen due to its high hydrogen density and liquid state under high pressure at room temperature. Currently, large‐scale industrial production of NH_3_ relies on the Haber‐Bosch process at high temperatures and pressures.^[^ ^129^ ^]^ The NRR has been proposed as a sustainable and carbon‐free method for NH_3_ production under mild conditions.^[^ ^130^ ^]^ Additionally, the electrochemical nitrate reduction reaction (NO_3_RR) has also attracted attention as a method to produce NH_3_ from nitrate, which simultaneously realizes the recycling of nitrogen‐containing pollutants.^[^ ^131^ ^]^ The key challenges with these reactions are the low reactivity and selectivity for NH_3_ due to the competition of the HER. Thus, highly efficient and economical electrocatalysts are essential. MXene‐supported SACs have been explored in these fields due to their excellent electrical conductivity, higher atomic utilization, and higher selectivity to adsorb specific ions.

**Figure 12:**
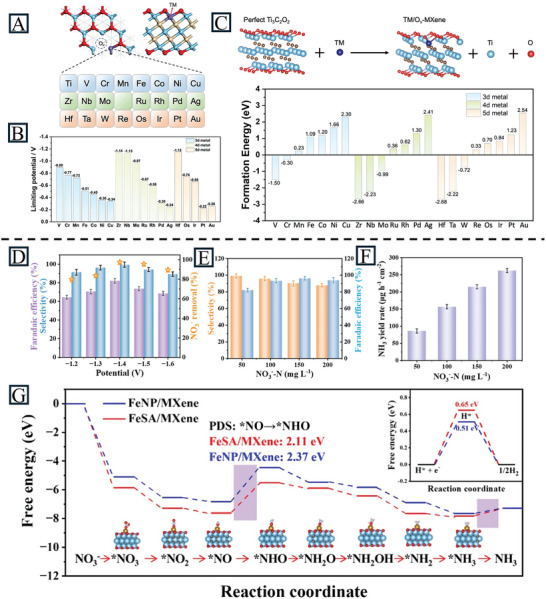
A) Top and side views of the atomic structure of TM/O_v_‐MXene. Atom labels: C (white), Ti (blue), O (red), and TM (purple). The screened TM atoms (from Ti to Au) are listed. B) Summary of limiting potentials on TM/O_v_‐MXene for NO_3_RR. C) Calculated formation energy of TM/O_v_‐MXene (TM = 3d to 5d transition metals). Reproduced with permission.^[^ ^132^ ^]^ Copyright 2023, Wiley‐VCH. D) Potential‐dependent Faradaic efficiency, NO_3_^−^ removal, and NH_3_ selectivity for Fe_SA_/MXene filter. E) Comparison of the highest Faradaic efficiency and NH_3_ selectivity for the Fe_SA_/MXene filter at different NO_3_^−^ concentrations. F) The NH_3_ yield rate of a Fe_SA_/MXene filter in 0.1 M Na_2_SO_4_ electrolyte with different concentrations of NO_3_^−^. G) Gibbs free energy diagrams of nitrate reduction to NH_3_ and H_2_ evolution reaction (the top right) over Fe_SA_/MXene and Fe_NP_/MXene. Reproduced with permission.^[^ ^135^ ^]^ Copyright 2023, American Chemical Society.

Many MXene‐supported materials are theoretically predicted to have a good NRR performance. For instance, Gao et al. explored the feasibility of SACs supported on Ti_3_C_2_O_2_ with oxygen vacancies for NRR applications.^[^ ^132^ ^]^ As shown in **Figure** **12**A, one O atom is removed from TM/MXene to form an O vacancy, and then the exposed Ti atoms are replaced by different TMs. With the DFT results, Pt/Ov‐MXene shows the lowest energy barrier to produce NH_3_, however, the HER outcompetes the NRR on this catalyst. Ag/O_v_‐MXene and Cu/O_v_‐MXene are the next best catalysts with a higher preference toward NH_3_ than hydrogen (Figure 12B). The formation energy *E*_form_ was calculated to evaluate the thermodynamic stability of the catalysts. The lower E_form_ of Ag/O_v_‐MXene (+2.3 eV) and Cu/O_v_‐MXene (+2.41 eV) were relative to that of Pt_SA_/O_v_‐MXene (+2.62 eV), meaning that these two catalysts should be thermodynamic stable (Figure 12C). Qu et al. reported a series of superior NRR catalysts by combining Ti_2_NO_2_ with 28 different SAs via first‐principles calculations. According to the formation energy calculation, most candidates are thermodynamic stable, while the elements with full or almost full electron shells (Zn, Ag, Cd, Au, and Hg) exhibit small formation energies. Thus, the MXene is a promising substrate to immobilize SACs.^[^ ^133^ ^]^ For the early transitional metals, the side‐on pattern is preferred where N_2_ would be horizontally anchored with both sides interacting with the substrate. However, as the number of d electrons increases to d^5^ or d^6^, the end‐on pattern is preferred where only one side of nitrogen coordinates with catalysts. For SAs/Ti_2_NO_2_, both the side‐on and end‐on patterns work due to the synergistic effects of SAs and Ti. With the enzymatic‐distal mechanism, the optimal performance was obtained in the d^8^ orbital system with Ni_SA_/Ti_2_NO_2_ (−0.13 V). Huang et al. designed 18 different SAs with two S‐functionalized MXenes (Ti_2_CS_2_ and Nb_2_CS_2_) and explored their activity toward the NRR.^[^ ^134^ ^]^ Compared to O‐functionalized MXenes (Nb_2_CO_2_ and Ti_2_CO_2_), the ΔG_*H_ of Nb_2_CS_2_ is large (0.8 eV), which indicates poor HER activity and the potential to be used for the NRR. Among all the candidates, Mo@Nb_2_CS_2_ is highly promising for its activity and selectivity. In the catalytic structure, Nb_2_CS_2_ acted as an electron donor during hydrogenation meanwhile MoS_2_ served as a medium to transfer electrons between the intermediate and the MXene. All these calculations and research provide a valuable reference for the future construction of SACs on MXenes.

To further explore MXene‐supported SACs for NRR applications, experimental investigations have also been conducted. Ren et al. reported an effective Fe_SA_/Ti_3_C_2_T_x_ catalyst for the NO_3_RR, which takes advantage of elevated activity, low nitrite selectivity of Fe and tunable electronic structure. It shows a higher NH_3_ Faradaic efficiency and selectivity (82.9% and 99.2%) than those of Fe_NP_/MXene (69.2% and 81.3%, Figure 12D–F). Here, the NO_3_^−^ is adsorbed onto Fe single atom sites to first produce *NO_3_, which then breaks down to *NO_2_ and *NO. The protonation reaction of *NO results in the generation of *NHO, *NH_2_O, and *NH_2_OH. The N‐O bonds in NH_2_OH are cleaved and protonated to form *NH_3_. Finally, the *NH_3_ is released from the surface of catalyst to produce NH_3_ (Figure 12G). The MXene acted as a support for Fe SAs with Fe‐O bonds and prevented them from corroding and leaching into the solution during the reaction.^[^ ^135^ ^]^ According to Table 2, MXene‐supported SACs exhibit significant promise for NRR applications, surpassing the performance of GO and MoS_2_.

## 5 Summary and Outlook

SACs are considered some of the most promising alternatives to traditional electrocatalysts due to their low cost and high efficiency. The simplified components and structures of SACs make it easier to identify the active sites and reveal the mechanism during electrochemical reactions. In this review, MXenes are introduced as potential support for SACs due to their unique properties, including large surface area, high electrical conductivity, good hydrophilicity, and tunable surface groups. We first discuss the impacts of synthesis and properties of MXenes for the introduction of SAs. The morphology and surface functional groups can be easily controlled by synthesis methods, benefiting the design of catalysts for specific applications. Moreover, the synthesis approaches of MXene‐supported SACs are involved which provide the anchoring sites in MXenes for SAs, including defect vacancy anchoring, metal‐support interactions, and selective atomic substitution. Advanced characterization techniques are frequently employed to validate the efficacy of SA doping. These methods, including STEM, XAS, XPS, and DFT, are instrumental in confirming the existence and coordination of SAs. Additionally, the applications of MXene‐supported SACs in electrocatalysis are discussed for the HER, OER, ORR, CO_2_RR, NRR, and NO_3_RR from experimental and theoretical perspectives. Many experimental and theoretical studies of MXene‐supported SACs in recent years have demonstrated their remarkable potential for electrocatalysis. In addition, the anchored SAs can be tailored according to the specific reaction from noble metal to transition metal atoms. However, the final performance of MXene‐supported SACs still needed to be improved. In order to fully realize the potential of MXene‐supported SACs, it is imperative to undertake further exploration in the following areas to overcome current obstacles, a task that poses challenges but is essential.

1. Enhancing current synthesis techniques and exploring novel approaches to produce single‐layer or few‐layer MXenes are crucial for practical applications. The prevailing methods primarily yield Ti‐based MXenes through in situ HF etching, resulting in single‐layer or few‐layer structures. However, MXenes with alternative functional groups like ‐Cl, ‐I, and ‐S pose challenges in exfoliation, limiting the surface area and active sites for SAs. Additionally, MXene surface groups influence substrate‐SA, thereby affecting catalytic performance. Hence, innovative approaches for generating single‐ or few‐layer MXenes with diverse terminal groups could enhance the potential of MXene‐supported SACs for high activity. To address this issue, we suggest that on the one hand, appropriate exfoliation techniques for MXenes (T = Cl, I, S) can be developed; and on the other hand, direct synthesis methods like CVD can be employed to precisely produce few‐layer or even single‐layer MXenes.
2. While various types of MXenes hold potential as substrates for SACs, research has predominantly focused on Ti‐based MXenes. Yet, other MXene variants, including Mo‐based, Nb‐based and V‐based, have demonstrated viability for MXene‐supported electrocatalysis. A comprehensive understanding of catalytic mechanisms associated with these MXene variants is pivotal for advancing MXene‐supported SACs. Besides exploring the catalytic performance of pristine MXene variants, the ability to anchor SAs and their impact on the activity of SAs can also be investigated by experimental and theoretical research.
3. The current MXene‐supported SACs suffer from insufficient loading of SAs. This is because the weak interaction between SAs and the substrate is not enough to overcome the strong surface energy of a single atom to prevent aggregation, resulting in low loading of SAs and limited catalytic performance. Hence, gaining deeper insights into modifying MXene substrates during the synthesis process to enhance SA loading is crucial for achieving highly catalytic MXene‐supported SACs. To realize high loading of SAs on MXenes, increasing the number of coordination sites on MXenes, such as enhancing surface areas or rich defects, can be effective. In addition, controlling the synthesis conditions such as reducing the temperature also helps to increase SA loading without agglomeration.
4. While theoretical investigations have highlighted the potential of MXene‐supported SACs for electrocatalytic applications, numerous assertions remain untested in experimental settings. Consequently, prioritizing experimental endeavors is imperative to fully actualize the industrial applications of MXene‐supported SACs. For the large number of theoretically predicted catalysts, the screening of them can be realized with high throughput computation and machine learning. Suitable experimental methods can then be employed to synthesize and analyze the target catalysts.
5. Despite some MXene‐supported SACs having exhibited outstanding electrochemical activity, their stability is still an unignorable issue that limits industrial applications. Currently, strategies to enhance the stability of MXene‐supported SACs mainly focus on two aspects. The first approach is to improve the stability of MXenes, which can be achieved through the following methods: a) modifying the surface functional group to a more inert one (halide or hydrocarbons) instead of ‐F, ‐OH, or ‐O; b) improving the quality of precursor MAX phase and etching it with non‐aqueous solvent; c) covering the MXene surface with an oxidation‐resistant layer. The second approach is to improve the stability of fixed SAs by enhancing the interaction between MXene and SAs or adjusting the coordination condition of SAs. However, achieving both high activity and good stability is challenging. There is a need to balance them during catalyst design and develop highly efficient and robust MXene‐supported SACs.

## Conflict of Interest

The authors declare no conflict of interest.
